# A computational system for Bayesian benchmark dose estimation of genomic data in BBMD

**DOI:** 10.1016/j.envint.2022.107135

**Published:** 2022-02-09

**Authors:** Chao Ji, Andrew Weissmann, Kan Shao

**Affiliations:** a Department of Environmental and Occupational Health, School of Public Health, Indiana University – Bloomington, Bloomington, IN 47405, USA; b DREAM Tech, LLC, Bloomington, IN 47401, USA

**Keywords:** Benchmark dose, Genomic data, Bayesian, BBMD

## Abstract

**Background:**

Existing studies have revealed that the benchmark dose (BMD) estimates from short-term in vivo transcriptomics studies can approximate those from long-term guideline toxicity assessments. Existing software applications follow this trend by analyzing omics data through the maximum likelihood estimation and choosing the “best” model for BMD estimates. However, this practice ignores the model uncertainty and may result in over-confident inferences and predictions, leading to an inadequate decision.

**Objective:**

By generally following the National Toxicology Program Approach to Genomic Dose-Response Modeling, we developed a web-based dose–response modeling and BMD estimation system, Bayesian BMD (BBMD), for genomic data to quantitatively address uncertainty from various sources. The performances of BBMD are compared with BMDExpress.

**Methods:**

The system is primarily based on the previously developed BBMD system and further developed in a genomic perspective. Bayesian model averaging method is applied to BMD estimation and pathways analyses. Generally, the system is unique regarding the flexibility in preparing/storing data and in characterizing uncertainties.

**Results:**

This system was tested and validated versus 24 previously published in-vivo microarray dose–response datasets (GSE45892) and 64 molecules data from the Open TG-Gates database. Short term transcriptional BMD values for the median pathway in BBMD are highly correlated with the long-term apical BMD values (R = 0.78–0.91). The BMD estimates obtained by BBMD were compared to those by BMDExpress. The results indicate that BBMD provides more adequate results in terms of less extreme values and no failure in BMD and BMDL calculations. Also, the pathway analysis in BBMD provides a conservative estimate because a broader confidence interval is established.

**Discussion:**

Overall, this study demonstrates that dose–response modeling using genomic data can play a substantial role in support of chemical risk assessment. BBMD represents a robust and user-friendly alternative for genomic dose–response data analysis with outstanding functionalities to quantify uncertainty from various sources.

## 1. Introduction

The benchmark dose (BMD) approach is a generally accepted approach for deriving a reference toxicity value of a chemical (Committee et al. 2017; Haber et al. 2018). Traditional BMD assessments typically utilizes pathological measures obtained from in vivo toxicological experiments, such as organ weight, to estimate the dose that can cause a critical change in response. Obtaining these pathological measures are often labor-intensive, time-consuming, and low throughput. Recently, the application of the cost-effective and high throughput microarrays in toxicogenomics shows its potential to lead the transformation from an observational science to one that involves using functional omics technologies for chemical risk assessments (NRC, 2007). Several studies have demonstrated that the transcriptional pathway-based BMD estimates from short-term in vivo transcriptomics studies e.g., 5 days exposure can effectively approximate the long-term apical BMD (Thomas et al. 2011; Thomas et al. 2012; Thomas et al. 2013a; Thomas et al. 2013b). Herein, to facilitate the implementation of BMD estimates for chemical risk assessment, a technically and scientifically reliable software is critically needed.

Existing genomic dose–response analysis software include BMDExpress (Phillips et al. 2019), BMDx (Serra et al. 2020) and FastBMD (Ewald et al. 2021). BMDExpress is a standalone application that was first developed in 2007 (Yang et al. 2007) and its recent stable version was developed by the National Toxicology Program (NTP) and now has been continuously updated and improved. BMDExpress uses core algorithms implemented in the US EPA’s BMDS (Davis et al. 2011) to analyze genomic dose–response data. BMDExpress is programed in Java and can be installed in any operating system. BMDx is an R/Shiny package that is published by scientists from Tampere University in 2020. FastBMD is a web-based tool written in R programming language and was developed by researchers from McGill University in 2021. In general, all the three software follow the main steps of NTP Approach to Genomic Dose-Response Modeling (NTP, 2018): (1) filtering gene expression data, (2) fitting BMD models, and (3) pathway enrichment analysis. These three systems are slightly different in gene filtering methods, model settings such as the choice of Benchmark Response (BMR), pathway annotations, and efficiency. Basically, the BMD calculations of these software utilize the maximum likelihood estimation (MLE) algorithms that the “best” model corresponding to the maximum likelihood is chosen for BMD estimates. However, this practice may underestimate the model uncertainty and consequently result in over-confident inferences and predictions (Clyde 2003).

An elegant way to address this dilemma is to use Bayesian model average (BMA) method (Shao and Gift 2014; Shao and Shapiro 2018). In the BMA model, the inference is based on the model averaged posterior rather than on a single model. Herein, in this paper, we utilized the BMA method to account for the model uncertainty and developed a web-based dose–response modeling and BMD estimation system for genomic data. The new system is primarily based on the framework of our previously developed BBMD system (Shao and Shapiro 2018), which outperforms the BMDS system in term of fewer failed BMD and BMDL calculations and estimates for a single continuous or dichotomous dataset. The system is available at the Bayesian BMD website (benchmarkdose.com). Besides, the genomic BBMD system provides a user-friendly option in data storage (Manual and Guidance, 2021). Different from the one-time running setting of other web-based genomic software applications, all the analyses through BBMD are automatically stored and accessible for future usage or sharing. Generally, the system represents a unique and state of the art methodology in the field of toxicogenomics.

In the next section, a detailed introduction on the genomic BBMD system is presented. In the third section, BBMD estimation results are compared with BMDExpress results using the 24 microarrays (Thomas et al. 2013b). To further validate the BBMD system, the pathway-based transcriptional biological effect points of departure (BEPOD) derived in the BBMD system is compared with the apical endpoint POD obtained from pathological changes in a short and long term using 24 previously published microarray dose–response datasets (Thomas et al. 2013b) and the in-vivo dose response datasets of 64 chemicals in the Open TG-Gates (Igarashi et al. 2015). Following this, a thorough discussion on the advantages and limitations of genomic BBMD system is presented. The paper is then concluded with future research directions.

## 2. The genomic Bayesian benchmark dose analysis system

### 2.1. Overview

The genomic BMD analysis module in BBMD system is developed to use functional omics technologies for chemical risk assessments. Being consistent with the other computational modules in BBMD, the genomic BMD analysis module try to streamline the modeling process while ensuring the scientific credibility of the analysis. The system uses a React application with Babel ES6 JavaScript as frontend and a python Django application with a PostgreSQL database as the backend. The frontend collects users’ inputs, and the backend is responsible for data management and analyses.

The flowchart for a genomic BMD analysis is demonstrated in the Supplemental Material Figure S1. Step by step guidance and examples of available data visualization can be found in the BBMD user manual^1^. The input data, such as the counts (e.g., RNA-seq) or intensities (e.g., microarray), prior to dose–response analysis, are supposed to be log transformed and normalized. In this study, the robust multi-array average normalization module in Affymetrix power tools is applied to quantile normalize raw CEL files. Once the normalized dataset is uploaded and processed by the system, the summary of inputs (e.g., doses level, number of samples, number of valid genes and invalid genes), the principal component analysis and density plots are shown for users to check the input data quality. The summaries give users an overview on the input dataset and identify and exclude inappropriate inputs for following analyses. The fold change and statistical tests, including one way ANOVA and trend test (Peddada et al. 2005; Williams 1971, 1972), are applied to identify a biologically plausible and statistically significant probe sets. These individual probe sets with a few statistics, including P-values, adjusted P-values, and fold changes, are displayed in a summary table. To help users quickly identify genes with the most meaningful changes, two volcano plots, one with unadjusted p-values and the other with adjusted p-values, are displayed. Given the pre-processing results, users can include or exclude specific genes to create several datasets for the BMD analysis. The prepared datasets along with the user selected models, BMR settings, and the MCMC (Markov Chain Monte Carlo) settings are input for the BMD analysis. These BMD estimates are then classified into significant pathways based on the well-established public gene ontology (GO) and pathway database e.g., KEGG (Kanehisa and Goto 2000), REACTOME (Fabregat et al. 2018), and GO panther (Mi et al. 2019). For each pathway, the BMDL-BMD-BMDU values for all genes that are associated with this pathway are plotted to help users visualize the distributions of pathway BMDs and individual genes’ BMDs. The datasets generated through the analysis processes are automatically stored on the BBMD server. Users can download these datasets at the end of the analysis or share with experts or peers.

### 2.2. Preprocessing

Metrics including P-values, adjusted P-values, and fold changes are applied to filter gene expression data. Fold change (*f*) is calculated using equation set (1). The default value of *f* is 2, which means that dose–response data with *f* smaller than 2 are filtered out. P-values are calculated by one way ANOVA and trend test. One way ANOVA is a well-known test to determine whether there are any statistically differences between the means of the experiment groups and the control group. In addition, we adapt William’s trend test (Williams 1971, 1972) and Oriogen (Peddada et al. 2005) to identify genes having a monotonical trend with respect to doses. That is, the maximum likelihood estimate (MLE) of mean response at i-th level is estimated by equation set (2) and the test statistic (*T*) is calculated by equation (3). Permutation and bootstrap methods are applied to calculate the probability (P-value) that *T_i_* (i-th permutation or bootstrap) is larger than *T*. With the P-values, adjusted P-values are calculated by the Benjamini-Hochberg methods to decrease the false discovery rate.

(1) $$f=\{\begin{array}{cc} absmax(-\frac{1}{{base}^{\left({\bar{x}}_{i}-{\bar{x}}_{0}\right)}}); & if\;{base}^{\left({\bar{x}}_{i}-{\bar{x}}_{0}\right)}<1\\ absmax({base}^{\left({\bar{x}}_{i}-{\bar{x}}_{0}\right)}); & if\;{base}^{\left({\bar{x}}_{i}-{\bar{x}}_{0}\right)}\geq 1 \end{array}$$

where ${\bar{x}}_{i}$ is the mean response at i-th dose level, ${\bar{x}}_{0}$ is the mean response at control level, and base is the log base of data log transformation.

(2) $${\hat{\mu }}_{i}=\{\begin{array}{cc} {max}_{1\leq u\leq i}{min}_{1\leq v\leq K}\frac{\sum_{j=u}^{v}n_{j}{\bar{X}}_{j}}{\sum_{j=u}^{v}n_{j}}; & if\;increasing\\ {min}_{1\leq u\leq i}{max}_{1\leq v\leq K}\frac{\sum_{j=u}^{v}n_{j}{\bar{X}}_{j}}{\sum_{j=u}^{v}n_{j}}; & if\;descreasing \end{array}$$

where ${\hat{\mu }}_{i}$ is the MLE of *μ_i_*, *K* is the number of dose levels, *i* is the index of the dose groups (*i* = 1, ⋯ ,*K*), *n_j_* is the number of samples at j-th level, and ${\bar{X}}_{j}$ is the mean response at j-th level.

(3) $$T=abs\max(\frac{{\hat{\mu }}_{i}-{\bar{X}}_{0}}{s\sqrt{\frac{1}{n_{i}}+\frac{1}{n_{0}}}})$$

where ${\bar{X}}_{0}$ is the mean response at the control level, *s* is an unbiased estimate of within group standard deviation, *n_i_* is the number of samples at i-th level, and *n*_0_ is the number of samples at control level.

### 2.3. Models

The genomic dose–response data are continuous individual data. Seven frequently used dose–response models are available in BBMD to quantify the relationship between response *y* and dose *x*. The details of the seven models and parameter ranges are given in the equations (S1) – (S7) of the Supplemental Material. Let *M_k_, θ_k_*(*k* = 1, ⋯ ,7) denote the seven models and model parameters respectively, and *π_k_* denotes the model weights. Then the *k*^th^ model explaining the response and dose data is *y* = *f_k_*(*x*|*θ_k_*). To fit this model, we assume the response *y* satisfies a normal distribution centered at *f_k_*(*x*|*θ_k_*) with standard derivation δ.


$$y|x_{i},M_{k},\theta_{k}∼Normal(f_{k}(x|\theta_{k}),\delta )$$

We apply Bayesian theorem and calculate the posterior distribution of the observed dataset (*x_i_,y_i_*). The posterior distribution is given by

$$p(\theta_{k}|y_{i})=p(y_{i}|x_{i},M_{k},\theta_{k})p(\theta_{k})$$

where *p*(*θ_k_*) is the prior distribution in the equations (S1) – (S7) of Supplemental Material and taken differently for each model. The posterior distribution is computed using MCMC sampling by PyStan (Carpenter et al. 2017).

In order to combine different models, we utilize the BMA. The BMD distribution *p*(*BMD*|*y_i_,x_i_,M_k_*) for each model is calculated using the posterior sample of model parameters. The posterior BMD distribution *p*(*BMD*|*y_i_,x_i_*) is given by summing the BMD distributions over different models weighted by model weights as

$$p(BMD|y_{i},x_{i})=\sum_{k}\pi (M_{k}|y_{i},x_{i})p(BMD|y_{i},X_{i},M_{k})$$

where the posterior model weights *π*(*M_k_*|*y_i_,x_i_*) are calculated by using Bayesian theorem as

$$\pi (M_{k}|X)=\frac{f(X|M_{k})\pi (M_{k})}{\sum_{k=1}^{K}f(X|M_{k})\pi (M_{k})},\mathrm{and}\;\sum_{k}\pi (M_{k}|X)=1$$

### 2.4. Benchmark dose estimation

A BMD/BMC (benchmark dose or concentration) is a dose or concentration that produces a predetermined change in the response. To calculate the BMD/BMC, a BMR needs to be defined. Two options for defining the BMR values are provided based on the central tendency: a) relative change and b) standard deviation shift, which are descried below.

f(BMD)±f(0)=relativechange×f(0)

f(BMD)±f(0)=k×standarddeviation,

where *f*(0) is the estimated response at zero dose, *f*(*BMD*) is the response at BMD, relative change (e.g., 10%) and *k* (e.g., 1) are values defined by user, and standard deviation is another parameter estimated in the model fitting process. As a note, for every model, every posterior sample has an estimate for *f*(0), standard deviation and an estimated BMD value. For a single model, the median, 5th percentile and 95th percentile of the posterior sample given the dose response data of probe set are named as BMD, BMDL and BMDU. With the posterior model weights *π*(*M_k_*|**X**), an integrated posterior distribution of BMD sample over all models can be established. The median, 5th percentile and 95th percentile values of the integrated posterior distribution is used to represent BMD, BMDL and BMDU of the model averaged estimates.

### 2.5. MCMC settings

The MCMC settings include MCMC iterations, number of Markov chains, warmup percent, and random seed. These settings may influence the running speed and the posterior sample converge. Based on our testing, empirical settings with one chain, 30,000 samples and 50% warm-up ratio are generally sufficient to ensure sampling convergence. These settings are consistent with the previously published BBMD system and a more detailed discussion on the settings can be found in (Shao and Shapiro 2018).

### 2.6. Pathway analyses

In the pathway analyses, platforms from Gene Expression Omnibus (GEO) are provided and users need to select the one associated with the uploaded genomic data. Four kinds of pathway analyses are provided to classify the BMA BMD analyses into significant pathways based on their NCBI Entrez Gene identifiers: a) Gene ID Analysis; b) GO Analysis; c) REACTOME Pathway Analysis; and d) KEGG pathway analysis. For each pathway analysis, the genes were matched with their associated categories, and the minimum, maximum, mean and median BMD were calculated for each category from the gene level fits of concentration-responsive genes within each category. Gene ID Analysis simply translates the probe set identifiers to NCBI’s Entrez Gene identifiers. GO Analysis utilizes ‘go-basis.obo’ and a python package GOATOOLS (Klopfenstein et al. 2018) to group the Entrez Gene identifiers into three sub-ontologies: biological process, cellular component, and molecular function. The ‘reactomepy’ python module is used to access the REACTOME database, and API request is used to access the KEGG database. For all the analyses, probe sets that measured more than one gene were removed from analyses. When different probe sets are associated with the same Entrez Gene identifiers, mean values of BMD are taken to represent the BMD value of the Entrez Gene identifiers. In order to determine whether the pathway is significant, P-values and percentages are calculated for each category. P-values are calculated based on Fisher’s exact two-tailed test by comparing the numbers of genes with BMD estimates with the numbers of genes without BMD estimates. For each category, percentage is defined as the ratio of the number of genes with BMD estimates that are on this category to the total number of genes that are related to this category. In the case study of this manuscript, pathways with P-value < 0.05, number of genes in the pathway > 1 and percentage ≥ 3% (Johnson et al. 2020) are defined as enriched pathways. The median BMD value of enriched pathways is defined as BEPOD and then compared with the POD which was derived from apical endpoints. Here, we use BMD estimates for comparison instead of using BMDL or BMDU because we want to focus on the correlation between genomic BMD and apical BMD and minimize the impacted from estimation uncertainty. Pearson’s correlation coefficient and the root-mean-square deviation (RMSD) are used to evaluate the correlation relationship and differences between BEPOD and POD. In order to capture the relative concordance of POD and BEPOD, the values being compared were transformed to the log scale with a base of 10 (Johnson et al. 2020).

## 3. BMD analysis comparison

### 3.1. Preprocessing comparison

**Fig. 1.**
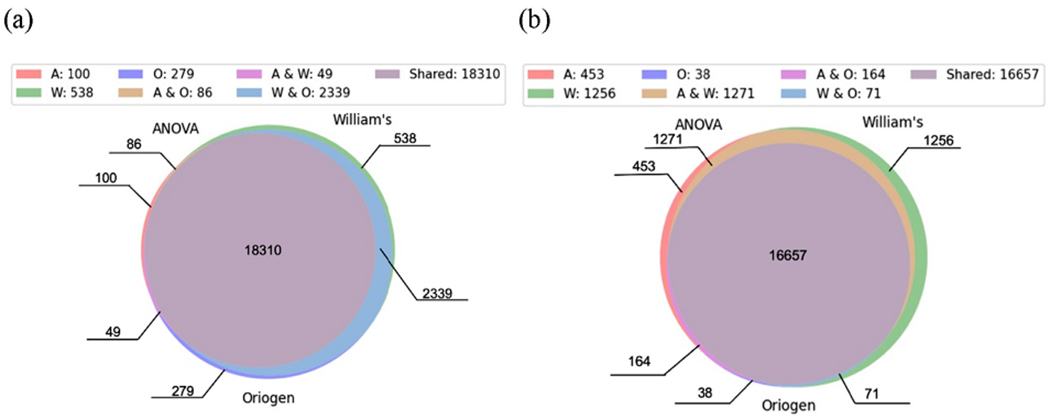
Venn diagram of the three preprocessing methods in BBMD (a) and in BMDExpress (b) (A: ANOVA, W: William’s, O: Oriogen, A&O: ANOVA & Oriogen shared; A&W: ANOVA & William’s shared; W&O: William’s & Oriogen shared; Shared: three shared).

The Venn diagram using three preprocessing methods in BBMD with same metrics is plotted in Fig. 1(a). For the 24 datasets, the trend tests, including Williams’ test and Oriogen, let more genes pass the preprocessing compared with one-way ANOVA. The genes pass one-way ANOVA can almost pass the trend tests as well. These three methods share around 99% common genes among the genes pass one-way ANOVA test (=18310/18545). The two trend tests produce ~ 98% similar genes.

The same datasets were loaded to BMDExpress for comparing the preprocessing results. One way ANOVA method in BBMD and BMDExpress produces exactly the same results. The total number of genes that pass the preprocessing was 18,545 in both applications. The Venn diagram of BMDExpress using these three preprocessing methods is plotted in Fig. 1(b). The two trend tests from BMDExpress generate slightly different results comparing with the counterparts from BBMD and share the majority common genes with the one-way ANOVA. The different hypotheses of the Oriogen method make the major contribution to the difference. In BBMD, the response and dose are assumed to be monotonic. In contrast, BMDExpress assumes the non-monotonic relationship between dose and response. In comparison, the preprocessing in BBMD generates a more consistent results among the three methods. The results of one-way ANOVA are used for further calculations and comparisons in this paper’s case studies as the results are the same in the two applications.

### 3.2. BMD estimates of single models

The 24 microarrays dose–response datasets (NCBI’s Gene Expression Omnibus: GSE45892) from Thomas et al. (2013b) were used to compare the performance of the two systems, BBMD and BMDExpress, under default settings. Details on the data and model settings are described in the Section 3 ‘Datasets’ of Supplemental Material. These data were collected from six toxicological studies in which adult rats (*Rattus norvegicus*) were exposed to one chemical at five dose levels for 5 days, 2, 4, and 13 weeks respectively. These six chemicals are 1,2,4-Tribromobenzene (TRBZ), Bromobenzene (BRBZ), 2,3,4,6-Tetrachlorophenol (TTCP), 4,4′-Methylenebis (N, N-dimethyl) benzenamine (MDMB), N-Nitrosodiphenylamine (NDPA) and Hydrazobenzene (HZBZ). For each dataset, 31,139 probe sets are tested. The datasets preprocessed by the one-way ANOVA method in the two systems were the same and then used for the BMD analysis. In total, 18,545 probe sets passed the one-way ANOVA test and were used as input for BMD analysis. One standard deviation BMR suggested by the NTP Approach to Genomic Dose-Response Modeling (NTP, 2018) was used in both systems for BMD calculation. The 18,545 BMDs (and associated BMDLs) values calculated using seven continuous models (i.e., the Linear, Power, Hill, Exponential 2, Exponential 3, Exponential 4 and Exponential 5 models) were compared. The comparison of the BMD estimates for single models are summarized in Table 1 and the quantities are explained below.

- Number of failed BMD or BMDL. The BMD estimates being reported as ‘not available’ or ‘error’ or ’< 0’ are regarded as ‘failure’. Typically, given limited number of dose levels, models with more parameters such as the Hill and Exponential 5 models are easier to fail or provide unrealistic estimates than other models. Because no failure was observed in BBMD, the failures in BMDExpress were not due to inappropriate BMR setting (e.g., the BMR is too large so that the fitted curve can never reach that level because of the plateau feature of these models). The failures were due to the computational reason that models with more parameters are more difficult to fit. Thus, a more elaborated algorithm such as MCMC that incorporates parameter priors makes the estimates more stable.
- BMD/BMDL ratio. The ratio is one of the US EPA criteria for judging appropriateness of models. Large ratios indicate greater uncertainty. The default setting for BMDExpress is that a BMD/BMDL ratio of > 20 results in a ‘questionable’ estimate. Here, these reported failed BMD or BMDL are removed for the BMD/BMDL ratio analysis. The median and the 95% confidence interval of BMD/BMDL ratio were reported.
- Number of reduced models, which only applies to BMDExpress. The BBMD system uses the MCMC posterior sampling method where all the parameters in a dose–response model are sampled in each iteration, so no parameter is reduced. However, in BMDExpress, the complex models with a power parameter may be reduced to a simpler model when power parameter hits the lower parameter bound at 1. This reduction may influence the BMD estimates.
- Comparisons between the two systems. The estimates of BMD and BMDL are compared using BMD ratios and BMDL ratios. Ratio is taken as the BMD (or BMDL) estimated in BBMD divided by BMD (or BMDL) estimated in BMDExpress. The Pearson’s correlation coefficients are used to determine the correlation of BMD (or BMDL) estimates in the two systems. For the ratios, all the failed BMD estimates are removed from analysis. The median and 2.5th – 97.5th percentile interval of ratio were presented. Because one default setting in BMDExpress is that the BMD value larger than the highest dose is removed for the category analysis, the correlation coefficients of the two systems is derived from the reasonable BMD estimates within the dose range. Linear regression was applied to fit the BMD (or BMDL) estimates for the two systems [BBMD (B) vs BMDExpress (E)]. BMD_B_-BMD_E_ and BMDL_B_-BMDL_E_ plots can be found in the Figures S2–S15 of Supplemental Material. The plots also exhibit a similar trend as the data shown in Table 1. The BMD_B_-BMD_E_ and BMDL_B_-BMDL_E_ estimates of Liner and Exp 2 models are overlapping. The BMD_B_-BMD_E_ and BMDL_B_-BMDL_E_ estimates of the Hill model show the highest divergences. In Figures S10–11, although the majority of data scatter around the regression line, a substantial part of data scatters below the regression line which indicates that the Hill model in BMDExpress produced more estimates that are significantly smaller (some closer to zero) than the counterpart from BBMD, especially the BMDL estimates. Hence, the 95% confidence interval of BMD to BMDL ratios and the BMDL estimates (as shown in Table 1) are more extreme than those from BBMD. The different algorithms in the two systems contribute to the differences. With the power parameter ≥ 1 restriction, around 18% (n = 3382) of the power parameter in the Hill model fitted to the 18,545 probe sets in BMDExpress are equal to 1, while in BBMD the power parameter is a distribution lower bounded by 1 (but never be a single value of 1). In addition to the model format simplification, unreliable BMDL estimation (resulting extremely low BMDL values) by the Hill model in BMDExpress is another factor causing the differences.

Overall, for the BMD analysis of a single probe set, BBMD has 0 failure but BMDExpress has143 failed BMD using the Hill model, 123 failed BMD using the Exp 5 model, and a few failures in other models. Models with more parameters have more failures than the simple ones with two parameters due to the parameter estimates algorithms. For the individual probe sets with successful BMD and BMDL estimates, the two systems give almost the same estimates using the Linear and Exp 2 model, and very similar estimates for the majority of the genes as suggested by the median values of the BMD ratios, BMDL ratios, and the scatter plots in the Figures S2–S5 of Supplemental Materials. The BMD_B_-BMD_E_ and BMDL_B_-BMDL_E_ plots of the Power model in Figures S8–S9 are evenly distributed. Although the majority of BMD and BMDL estimates in Figures S6–S7 and S10–15 for the Exp4, Hill, Exp3, and Exp 5 models are well correlated across the two systems, there are some notable exceptions. The BMD(L) estimates of Exp 4 and Exp 5 models (Figure S6–S7 and S14–S15) in BMDExpress have more extreme estimates for probe sets where BBMD gives a relatively small estimate. The other exception is that the Hill model in BMDExpress (Figures S10–S11) has some extreme estimates for probe sets where BBMD gives a relatively large estimate. The BMD estimates of the Exp 3 model in Figure S12 are evenly distributed, but a disparity exists in BMDL estimates where BMDExpress estimates are close to zero. The differences are primarily due to different algorithms are used for parameter estimates. The other important reason that causes the difference in median ratios is that the model fitting algorithm used in the BMDExpress system may reduce models with more than two parameters to a simplified format. For example, the power parameter in the Power model hit the lower bound of the parameter restriction and became 1, (i.e., the Power model is essentially the Linear model) for 9,845 out of the 18,545 probe sets tested. Additionally, for models with three parameters or more (especially the Exp 4, Exp 5 and Hill models), BMDExpress produces more extreme values of BMD/BMDL ratio while the median values of the ratios in the two systems are quite similar. As shown in Table 1, the 95% confidence interval of BMD/BMDL ratio for the Hill, Exp 4 and Exp 5 models in BMDExpress are much wider than the counterparts estimated from BBMD. The comparison of BMD and BMDL estimates produced by these two systems [BBMD (B) vs BMDExpress (E)] through the BMD_B_/BMD_E_ and BMDL_B_/BMDL_E_ ratios also demonstrates that the BMDExpress generates some extreme BMD and BMDL values by the Hill, Exp 4 and Exp 5 models that make the upper bound of BMDLs ratio very large. In contrast, BBMD is more stable in terms of more plausible lower bound estimates.

### 3.3. BMA vs. Best model estimates

BMDExpress uses the estimates from the best model as the final BMD and BMDL for each probe set (the default setting of ‘best model’ selection is to select the next best model with P-value > 0.05 when the best model is the Hill model), while BBMD uses the Bayesian model-averaged BMD and BMDL estimates that takes estimates from all selected models into considerations. The degree of each single model that contributes to the model-averaged BMD(L) estimates is primarily determined by the model fitting quality of each model. If the model explains the data poorly, the weight of this model may be close to 0. Hench, the model averaged BMD estimates in BBMD are more plausible by taking model uncertainty into account. To reveal the differences between these two methods, a few important statistics obtained from BBMD and BMDExpress were summarized and compared in Table 2, including the median and the percentile interval of BMD/BMDL, BMDU/BMDL, and BMDU/BMD ratios. The correlations of BMD and BMDL estimates from the two systems and the ratios are presented. The BMD_B_-BMD_E_ and BMDL_B_-BMDL_E_ plots of MA v.s. Best can be found in Figures S16–19 of the Supplemental Material. Generally, the median values of these metrics are similar, but the 95% confidence bounds of these metrics in BMDExpress are much broader than the ones in BBMD mainly because of the extreme lower and upper bound estimates from BMDExpress.

The best model is typically the one with the lowest AIC (Akaike Information Criterion) value which is mainly determined by the model’s log-likelihood estimates and number of parameters. The reliability and plausibility of the BMDL estimates do not have direct impact on model selection, so some of the extreme lower and upper bound estimates are passed on to the best model BMD and BMDL estimates, as indicated by the 95% confidence intervals of BMD/BMDL, BMDU/BMDL and BMDU/BMD ratios in Table 2, as well as the scatter plots of Figures S16–19 in the Supplemental Materials. On the other hand, the intervals of the ratios based on BMA estimates from BBMD are within a more reasonable range. After removing probe sets with BMD estimates greater than the max dosage, the BMD and BMDL estimates in two systems are highly correlated, and the medians of BMD and BMDL ratio are close to 1. There is no clear patten in the BMD estimates from the BMA and best model approaches, but the best model BMDL estimates are generally larger than the BMA BMDL estimates (as the slope of the linear regression is>1) and this is probably because the BMA method takes model uncertainty into account.

In some alternative applications, user can choose to filter the BMD results prior to pathway analysis with the purpose of exclude extreme BMD estimates (either too large or too small) to make the BMD results more plausible for subsequential pathway BMD analysis. Such filters are not implemented in BBMD because some important features of the BBMD system have substantially reduced the chances that implausible BMDs being produced. First, the filters in the data pre-processing step screened out the gene(s) that do not show a clear dose–response trend, accordingly, extremely large BMD estimates caused by flat dose–response curves have been avoided; Second, as we demonstrated in this paper and a previous study (i.e., Shao and Shapiro 2018), BBMD is more robust in calculating BMD and BMDL (i.e., fewer failed or extremely low BMDL estimates) than the BMDExpress which basically uses the computational module in EPA’s BMDS for BMD estimation. Therefore, a filter for BMD estimates has not been implemented in BBMD, but the performance in analyzing various genomic datasets will be continuously monitor to guide future development of the BBMD.

## 4. Pathway analysis comparisons

### 4.1. Cumulative distribution curve comparision

**Fig. 2.**
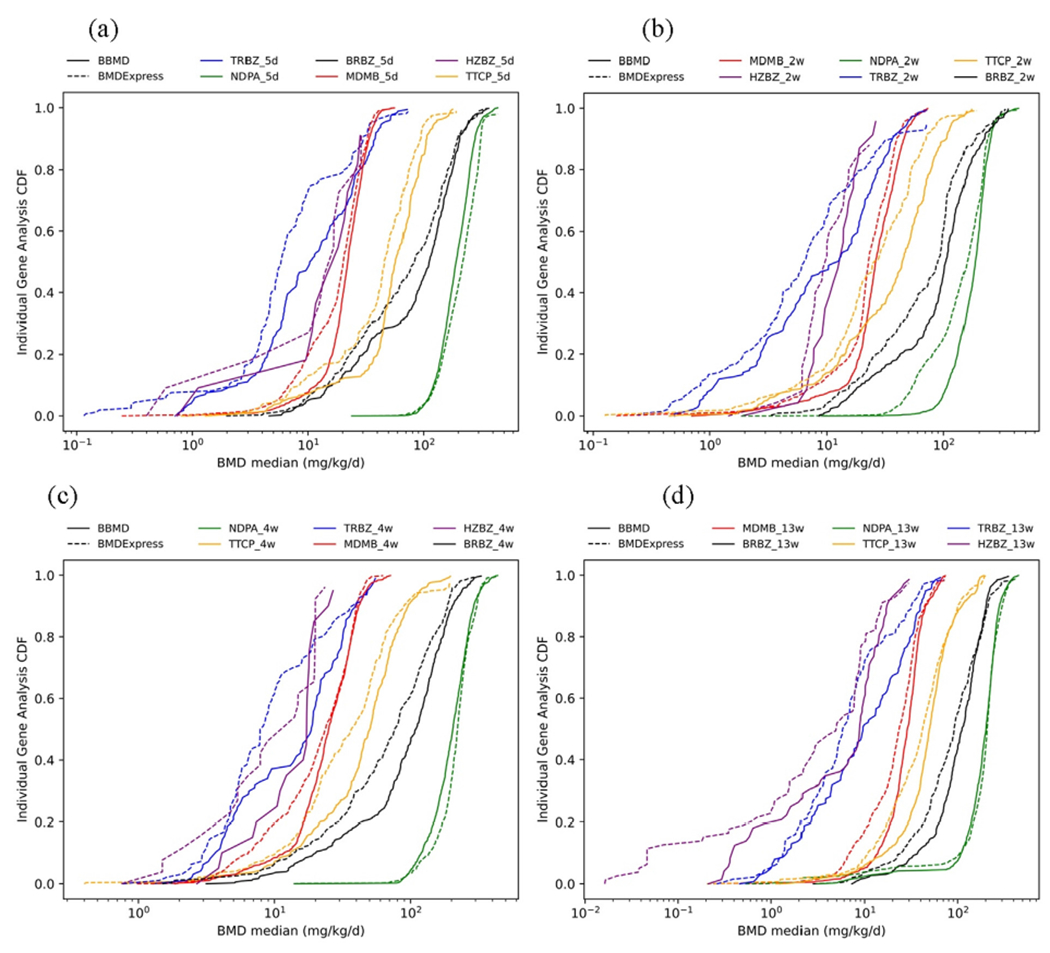
Cumulative distribution comparison of individual gene analysis for BRBZ, NDPA, TTCP, MDMB, TRBZ and HZBZ at (a) 5 days, (b) 2 weeks, (c) 4 weeks, and (d) 13 weeks (dashed lines are values from BMDExpress and solid lines represent values of BBMD).

**Fig. 3.**
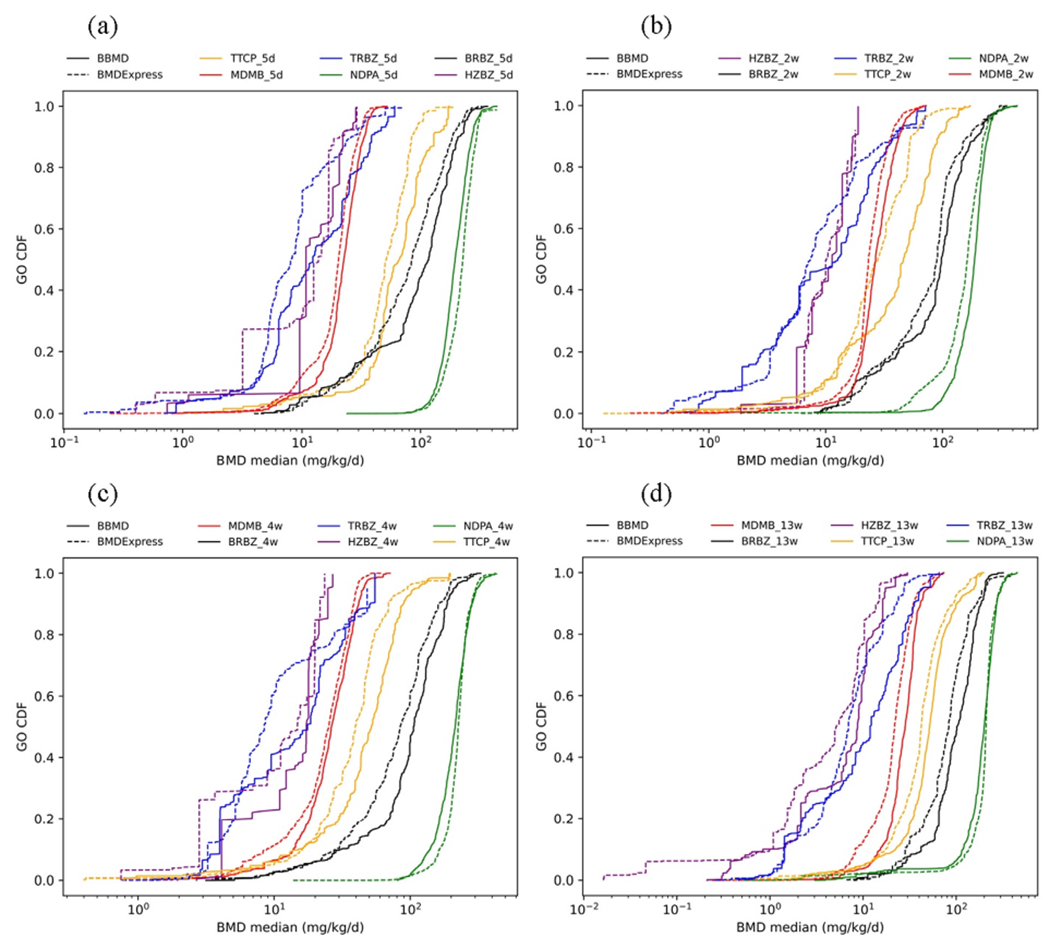
Cumulative distribution comparison of GO analysis for BRBZ, NDPA, TTCP, MDMB, TRBZ and HZBZ at (a) 5 days, (b) 2 weeks, (c) 4 weeks, and (d) 13 weeks (dashed lines are values from BMDExpress and solid lines represent values of BBMD).

**Fig. 4.**
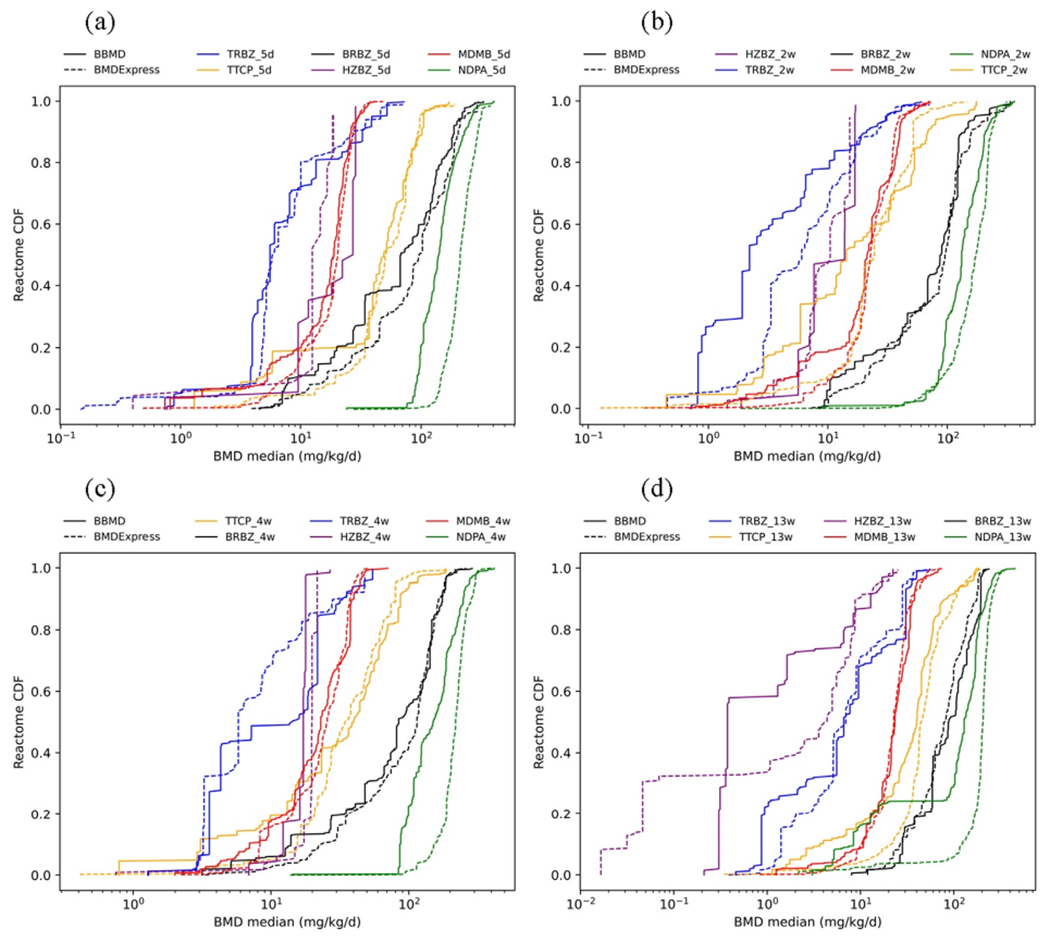
Cumulative distribution comparison of REACTOME pathway analysis for BRBZ, NDPA, TTCP, MDMB, TRBZ and HZBZ at (a) 5 days, (b) 2 weeks, (c) 4 weeks, and (d) 13 weeks (dashed lines are values from BMDExpress and solid lines represent values of BBMD).

There are three shared pathway analysis methods in BBMD and BMDExpress: individual gene analysis, GO analysis, and REACTOME pathway analysis. To examine how different the BMDExpress system (MLE algorithm and best model method) and the BBMD system (MCMC algorithm and BMA method) are in pathway analysis, the cumulative distribution plots of the BMD median of the 24 datasets using these three methods are shown in Figs. 2–4. In general, the dashed cumulative distribution curves (CDCs) based on BMDExpress estimates are similar to the solid CDCs based on BBMD estimates, indicating that the two systems provide similar pathway analysis results. The pathway analysis results from the two systems are more inconsistent for the chemicals TRBZ and HZBZ both of which have relatively low BMD estimates. These plots also illustrate that, in most cases, the dashed CDCs are slightly to the left of the solid CDCs, indicating that pathway BMDs estimated from BMDExpress are generally slightly smaller the counterpart from BBMD. To better visualize the differences of sensitive pathways where the lower tail of BMD is in the pathway level, the CDCs in which the x and y axes are on log-scale are shown in Figures S24–S26 in the Supplemental Material. The lower tail follows a similar tendency of the overall distribution that was described before. The median values of BMDs, BMDLs and BMDUs of all these pathways for the three common pathway analyses methods in BBMD and BMDExpress are compared and summarized in Table 3. As a note, the GO pathway analysis only uses the GO pathways of biological process for both systems. The apical BMD values calculated by BMDS and BBMD are also provided in Table 3. The data and the model settings for apical POD calculations are described in the Section 4 ‘Modeling setting’ of the Supplemental Material. To better visualize these values, ranges of the apical POD and BEPOD are shown in Figures S20 to S24 in the Supplemental Material.

In our study, we use the median pathway BMD as the BEPOD. Under this setting, the BMDL-BMD-BMDU ranges in BBMD are generally wider than the ranges in BMDExpress as indicated by results in Table 3. The BMDL values in BBMD are either close to or lower than values in BMDExpress, and the BMDU values in BBMD are either close to or higher than values in BMDExpress. The broader confidence bound may be due to the considerations of model uncertainty in BMA as the BMDL and BMDU values account for uncertainty in dose–response estimates. As a note, the BMD values in both systems are reasonable and are highly related to the apical POD values.

### 4.2. POD and BBMD-derived BEPOD correlation analysis

The previously discussed 24 microarrays only tested 6 chemicals. Among the 6 chemicals, HZBZ has no adverse noncancer pathological changes for short-term experiments and thus are removed for the correlation analysis. To better reveal the relationship between BEPOD and POD, in this analysis, the 24 microarray from Thomas et al. (2013b) and the in-vivo genomic dose–response data of 64 molecules from the Open TG-Gates database are used to conduct the correlation analysis (Igarashi et al. 2015). Raw CEL data were downloaded from the Open TG-Gates database. Then the data were quantile normalized using the multi-array average normalization module from the Affymetrix power tools. Then, the normalized data were input to BBMD for pathway calculations under the default settings. The corresponding PODs of each chemicals were also collected from Thomas et al. (2013b) and Johnson et al. (2020). In Thomas et al. (2013b), PODs for noncancer effects of the target tissues were available at different exposure durations from 5 day to 13 weeks. In Johnson et al. (2020), the PODs were generated for the apical endpoint with a treatment-related change at 29 days of exposure. Here the median BMD of all transcriptional GO-BP pathway was calculated to represent the BEPOD. The correlation between the POD and BEPOD was estimated using the Pearson’s correlation coefficient. Results indicate that the BEPOD of each molecule and POD are highly correlated. That is, the molecular changes caused by chemical exposures in a short term are highly associated with symptoms of disease in a long term. An empirical ratio factor, 0.19, was found by linear regression between BEPOD and POD. It is important to note that the ratio does not affect the correlation relationship but only affects the differences between PODs and BEPODs. The ratio factor was applied to the scatterplots in the non-cancer apical points section.

#### Non-cancer apical points

**Fig. 5.**
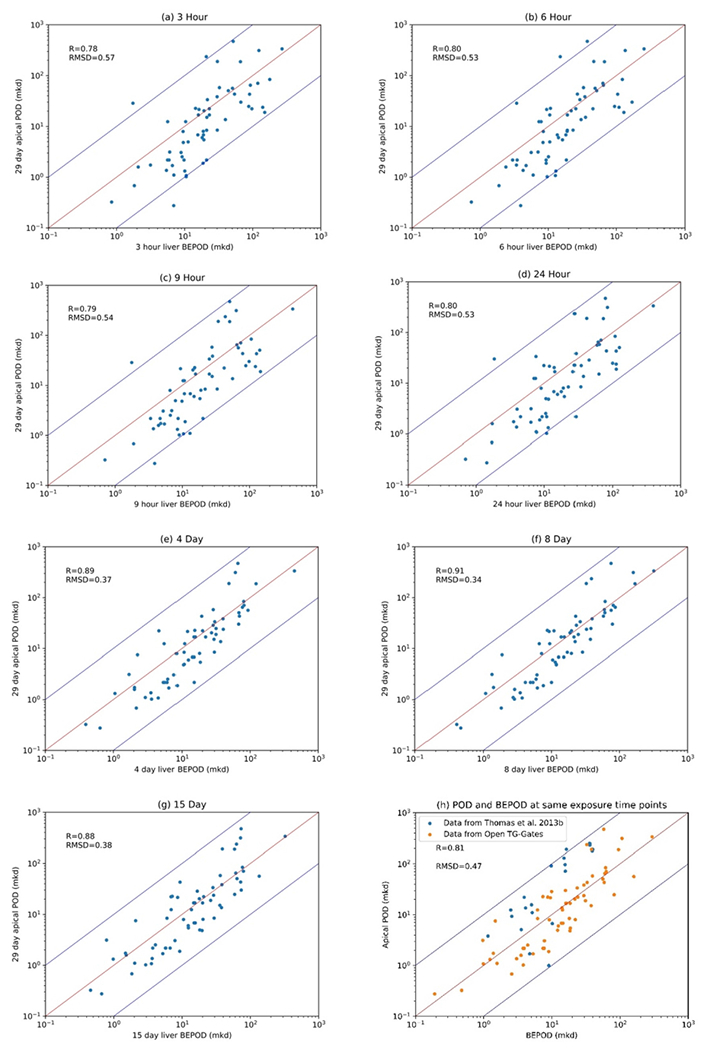
Scatter plots of the relationship between POD and BEPOD at different exposure time points from 3 h to 13 weeks. The red line represents x = y, and the blue lines represent 10-fold difference between the BMD values. (a)-(g) are the scatterplots of BEPOD and POD at 3-hour, 6-hour, 9-hour, 24-hour, 4-day, 8-day, and-15 day respectively. (h) is the scatterplot of BEPOD and POD at the same exposure time points. The blue dots are data from (Thomas et al. 2013b) that the BEPOD and apical POD are both at 5 day, 2 weeks, 4 weeks and 13 weeks. The orange dots are data from Open TG-Gates (Igarashi et al. 2015) that the BEPOD and apical POD are both at 29 day. (For interpretation of the references to colour in this figure legend, the reader is referred to the web version of this article.)

For the non-cancer apical points, the scatterplots of POD and BEPOD for different or same exposure durations are displayed in Fig. 5. The data of each molecule are listed in Table S2 BMD values for chemicals in Fig. 5 of the Supplemental Material. A positive correlation was observed between the apical POD and the BEPOD across all exposure durations. The R values ranged from 0.78 to 0.91 and the RMSD ranged from 0.34 to 0.57. The higher the Pearson’s correlation coefficient value, the lower the RMSD values. The BEPOD derived from subacute exposure experiments (3 h – 24 h) is highly correlated with the apical POD from subchronic experiments (4 day – 13 week). This correlation relationship supports the application of this short-term in-vivo toxicogenomic study to prefilter chemicals for pathological toxicology experiments. In Johnson et al. (2020), data were grouped into consistent and inconsistent dose levels as 22 molecules among the 64 molecules have inconsistent dose levels. Typically, the correlation between BEPOD and POD in the inconsistent dose levels group is lower than the consistent level group. Here, to simplify the comparisons, only the exposure duration was used to classify the data.

#### Cancer apical points

**Fig. 6.**
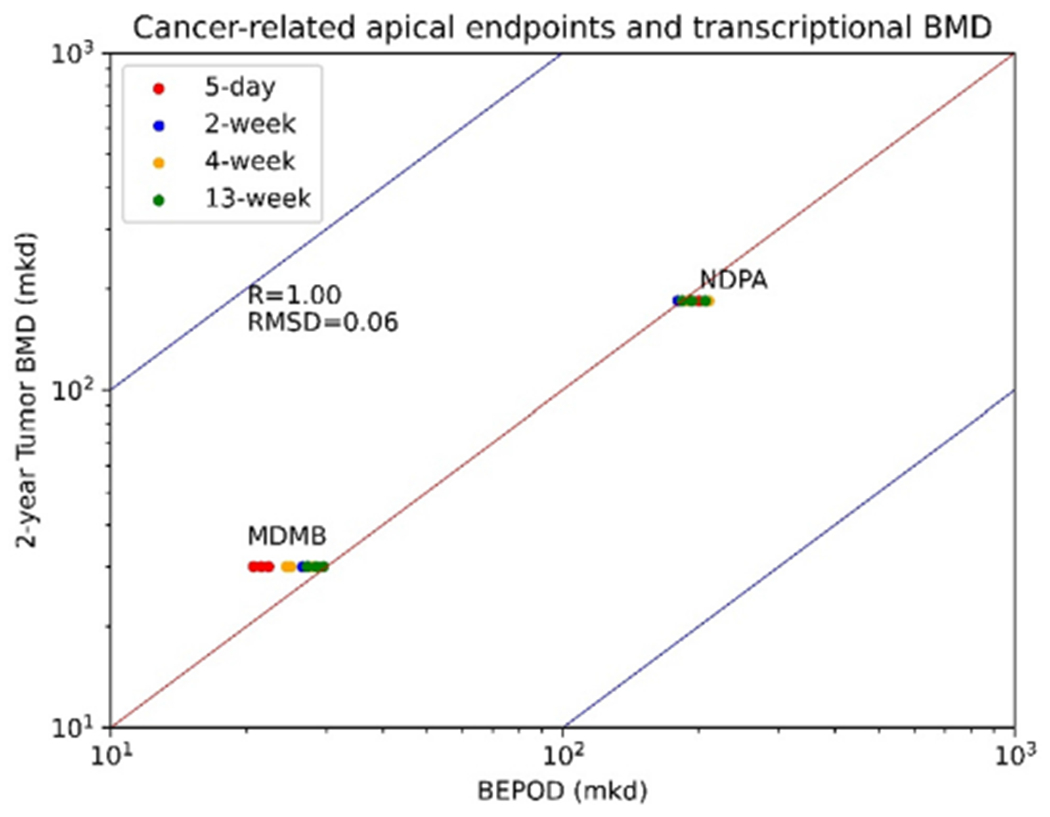
Scatter plots of the relationship between POD and BEPOD for cancer-related apical endpoints. The blue line represents x = y, and the red lines represent 10-fold difference between the BMD values. (For interpretation of the references to colour in this figure legend, the reader is referred to the web version of this article.)

Among the six chemicals in Thomas et al. (2013b), MDMB and NDPA are related to the tumor incidence. To reveal the correlation relationship between cancer-related endpoints and the transcriptional BMD, cancer-related apical endpoints derived from the 2-year rodent cancer bioassays (Thomas et al. 2013b) are compared with the transcriptional BMD values (Fig. 6). Same as the non-cancer apical points, the median values of the transcriptional BMD values were regarded as the BEPOD. GO-BP, Reactome and KEGG pathway classification methods were applied to derive the BEPOD. With the limited number of chemicals, the POD and BEPOD are highly correlated (r = 1.0) and the RMSD is 0.06. Results were not too different among the three pathways methods.

## 5. Discussion

BBMD offers a number of important advantages in modeling genomic dose–response data for BMD estimation, including more reliable BMD estimation (e.g., more plausible lower and upper bound) based on the MCMC algorithm and more comprehensive quantification of uncertainties (e.g., model uncertainty). On the other hand, the BBMD system is also limited by its computational speed, because the full-scale MCMC posterior sampling is slower than MLE optimization. For each probe set, BBMD currently takes about 6 s to complete the analysis under the default setting, while BMDExpress takes roughly 0.1 s to finish the calculation. To overcome the weakness, the BBMD is designed to allow users to process and analyze the data in the background, i.e., no close attention is needed, and users will be notified once the analyses are completed. Additionally, the hardware configuration of the server will be continuously improved if budget allows. Second, the present BBMD system only applies for the modeling of in-vivo transcriptional dose–response data but not for in-vitro transcriptional data. Because of the considerable uncertainty in in-vitro to in-vivo extrapolation (IVIVE) modeling (Auerbach and Paules 2018), incorporating the IVIVE modeling to BBMD and using BBMD to assimilate in vitro transcriptional data has not been implemented in the BBMD system and will be a future research direction. Finally, the toxicogenomic study typically reports different toxicological effects and endpoints with time stamps, and the BMD estimates may also depend on exposure duration and time point. Further research will be conducted on the feasibility of extending BBMD to model time-dependent transcriptional data.

Generally speaking, the BMDExpress and BBMD provide reasonably close results regarding single model BMD estimation and pathway BMD analysis while smaller distinctions exist. In Thomas et al. (2013b), the BMD values from the most sensitive pathway (the lowest BMD values) are used as BEPOD, but we use the median BMD values in the BBMD system. Our study suggests that the median pathway value is more stable across the short-term in-vivo studies and has a slightly higher correlation coefficient and a slightly lower RMSD value (Johnson et al. 2020) when compared with the POD. For example, the r values in (Johnson et al. 2020) between BEPOD-POD for days 4 and 8 are 0.65–0.85, but here we have r = 0.89–0.91 for days 4 and 8. The other differences when compare with the results in Thomas et al. (2013b) may result from the choices of BMR and the pathway database. In their study, 1.349 standard deviation in the control animals was used as BMR and we used 1 standard deviation in the control group instead. The 1 standard deviation follows the NTP’s guidance on genomic dose–response modeling. The other reason contributes to the difference is that the Gene Go Metacore database is used for pathway analysis in their study (Thomas et al. 2013b), but the publicly available pathway databases are used in our study.

Overall, this paper presents a probabilistic Bayesian modeling system BBMD for the genomic dose–response modeling. BBMD generally follows the NTP Approach to Genomic Dose-Response Modeling, and BBMD is unique regarding the flexibility in preparing and storing data as well as in providing model averaged BMD estimates considering model uncertainty. BBMD was tested and validated versus 24 previously published microarray dose–response datasets and the 64 molecules data from Open TG-Gates. The median values of significant BBMD pathways are highly correlated with the cancer (r = 1) and non-cancer related apical BMD values (r = 0.78–0.91). Comparing the BMD analysis in BBMD and BMDExpress system, BBMD provides a more stable estimate with no failure and less extreme estimates. As BBMD accounts for model uncertainty, the confidence intervals of pathway analysis in BBMD are broader than the ones in BMDExpress. That is, BBMD provides a relatively conservative estimate. Overall, this study demonstrates the feasibility of utilizing the in-vivo genomic data for chemical risk assessment in a Bayesian framework.

## 6. Conclusion

BBMD represents a user-friendly alternative for genomic dose–response data analysis with advantages in quantifying uncertainty. The main contributions of this work are extending the BBMD system to process genomic data and support the hypothesis that a short-term transcriptome based BEPOD has a strong positive correlation with the longer-term apical endpoint POD. The future developments of BBMD include the incorporation of IVIVE model to assimilate the in-vitro transcriptional genomic data. Also, further research on modeling the time-dependent genomic data can be conducted.

## Supplementary Material

1

2

## Figures and Tables

**Table 1:** Comparison of BMD/BMDL Estimations for Genomic Data.

Quantities measured	Linear	Power	Hill	Exponential 2	Exponential 3	Exponential 4	Exponential 5
**BMDExpress**							
Number of failed BMD	2	0	143	40	3	87	123
Number of failed BMDL	0	3	387	136	256	0	5
BMD/BMDL ratio (median)	1.341	1.443	1.855	1.337	1.490	1.683	1.879
95%	1.251–1.987	1.253–1.986	1.13–9.20E + 04	1.228–2.048	1.237–6.813	1.264–325.224	1.202–312.043
Number of reduced models (total: 18545)	NA	9845 to linear	NA (3382 power parameter = 1)	NA	9845 to exp2	3990 to exp2	4735 to exp3/4
**BBMD**							
Number of failed BMD	0	0	0	0	0	0	0
Number of failed BMDL	0	0	0	0	0	0	0
BMD/BMDL ratio (median)	1.363	1.546	1.705	1.357	1.535	1.800	1.803
95%	1.267–2.002	1.198–1.930	1.213–8.188	1.243–2.038	1.197–1.975	1.295–15.222	1.181–30.418
**Comparison**							
Correlation coefficient for BMD	1.000	0.969	0.898	0.997	0.955	0.943	0.942
Correlation coefficient for BMDL	0.999	0.982	0.856	0.999	0.910	0.809	0.873
Ratio of BMDs (median)	1.064	1.357	1.164	1.064	1.673	1.007	1.057
95%	1.028–1.070	0.983–1.938	0.832–81.383	1.015–1.072	0.941–2.348	0.224–1.245	0.107–1.941
Ratio of BMDLs (median)	1.047	1.285	1.279	1.047	1.326	0.970	1.071
95%	1.023–1.056	1.059–1.686	0.695–7.52E + 06	1.012–1.057	1.080–9.034	0.088–85.091	0.054–112.744

**Table 2:** Comparison of MA and Best Model Estimations for Genomic Data.

Quantities measured	BMDExpress (Best)	BBMD (MA)	Comparison	Values
BMD/BMDL ratio (median)	1.416	1.695	Correlation coefficient for BMD	0.326 (0.936)*
95%	1.145–364.420	1.254–25.066	Correlation coefficient for BMDL	0.893 (0.9)*
BMDU/BMDL ratio (median)	2.150	3.023	Ratio of BMDs (median)	1.089
95%	1.284–2.602e + 07	1.465–199.052	95%	0.603–4.962
BMDU/BMD ratio (median)	1.495	1.831	Ratio of BMDLs (median)	0.952
95%	1.056–305834.219	1.136–24.351	95%	0.341–113.182

* Values out of the parentheses are the correlations calculated by all BMD (BMDL) values, and values in the parentheses and values in the parentheses are the correlations calculated by removing BMD > max dos.

**Table 3:** Comparison of MA and Best Model Estimations for Genomic Data.

Chemical	BMDS*			BBMD			BBMD GO pathway			BMDExpress GO pathway		
5 days	BMD	BMDL	BMDU	BMD	BMDL	BMDU	BMD	BMDL	BMDU	BMD	BMDL	BMDU
TRBZ	12.398	5.489	27.198	26.014	11.509	33.326	13.182	5.364	36.077	7.807	3.798	16.588
BRBZ	191.000	159.000	207.336	172.125	121.084	208.457	86.245	36.211	167.892	85.622	59.551	145.476
TTCP	94.763	52.986	99.500	81.941	57.644	132.545	51.628	33.880	92.587	49.611	36.156	96.184
MDMB	22.100	13.700	25.073	26.088	18.551	32.020	20.713	13.203	34.816	20.413	14.496	31.064
NDPA	164.767	119.014	202.605	196.604	110.082	254.972	190.315	115.218	307.528	226.298	164.197	308.205
2 weeks												
TRBZ	8.659	5.948	9.894	7.900	5.169	10.621	13.643	5.607	42.809	7.675	4.038	15.870
BRBZ	165.354	125.238	273.228	159.597	112.893	233.489	79.573	34.799	146.484	90.110	53.474	143.319
TTCP	13.565	7.998	29.146	17.318	10.256	37.082	21.744	7.452	96.155	25.688	13.437	58.304
MDMB	13.848	4.800	15.930	9.775	4.529	13.905	26.580	13.947	49.809	23.282	17.180	36.286
NDPA	304.065	303.547	320.091	307.919	257.874	326.474	189.478	92.191	305.620	163.560	114.900	247.314
4 weeks												
TRBZ	4.562	2.669	5.692	5.091	3.270	6.997	18.764	3.465	40.210	7.791	4.001	16.654
BRBZ	72.000	40.280	120.536	119.992	55.025	165.221	83.049	22.670	164.911	83.755	46.514	144.682
TTCP	10.290	7.279	15.004	11.587	5.252	25.708	53.635	28.992	101.201	39.743	25.856	61.387
MDMB	3.350	0.501	3.989	1.223	0.346	3.450	25.017	16.100	41.715	25.398	17.980	36.446
NDPA	190.818	119.014	202.605	171.945	119.219	206.279	210.345	120.723	304.773	226.135	169.799	299.347
Chemical	BMDS*			BBMD			BBMD GO pathway			BMDExpress GO pathway		
		
13 weeks												

TRBZ	3.757	2.013	10.298	4.713	1.985	19.570	6.105	2.247	29.985	6.946	3.561	15.317
BRBZ	130.486	110.262	166.806	131.368	95.842	167.125	82.501	40.213	166.701	83.379	56.968	140.704
TTCP	1.517	1.093	2.255	1.131	0.581	4.471	47.595	28.769	94.863	42.111	32.018	65.640
MDMB	15.817	6.282	17.350	11.582	5.535	15.854	27.203	13.635	52.840	21.846	15.529	37.305
NDPA	134.059	131.165	159.538	154.993	105.416	196.360	206.495	128.774	298.882	209.629	151.125	271.620

Chemical	BBMD Reactome pathway			BMDExpress Reactome pathway			BBMD Individual gene			BMDExpress Individual pathway			KEGG pathway		
5 days	BMD	BMDL	BMDU	BMD	BMDL	BMDU	BMD	BMDL	BMDU	BMD	BMDL	BMDU	BMD	BMDL	BMDU
TRBZ	9.600	4.030	25.050	5.778	3.268	11.398	10.135	3.720	34.700	5.792	3.336	11.464	12.760	4.420	45.840
BRBZ	119.000	50.320	213.490	102.533	61.230	157.280	111.670	48.610	214.560	83.341	58.224	140.386	105.970	44.600	195.950
TTCP	54.000	33.330	105.250	50.104	38.270	93.693	57.220	34.680	133.990	46.135	35.362	72.744	60.855	33.180	133.085
MDMB	22.352	12.998	37.342	20.289	14.352	29.947	22.142	12.847	36.957	20.263	14.499	30.236	21.524	11.178	36.434
NDPA	183.736	112.897	299.323	211.920	157.110	291.603	190.922	114.914	305.877	215.653	154.335	303.061	200.286	117.901	313.915
2 weeks															
TRBZ	5.520	2.860	9.445	6.043	3.124	11.018	13.230	3.300	33.170	6.008	2.917	14.305	7.190	2.380	44.140
BRBZ	104.185	48.190	186.120	97.747	66.068	156.145	104.150	43.040	195.140	89.203	50.612	136.531	92.030	34.920	173.230
TTCP	29.325	7.450	123.140	24.484	12.583	76.178	44.085	12.715	96.155	24.585	12.776	58.304	48.460	8.360	123.140
MDMB	27.129	15.043	54.440	22.860	17.103	35.235	26.262	14.215	51.221	22.708	16.944	35.966	27.420	14.717	54.077
NDPA	179.850	93.501	270.550	171.896	120.796	238.441	185.362	84.803	306.427	157.619	110.847	235.663	192.561	82.860	320.638
4 weeks															
TRBZ	17.515	3.965	34.180	5.778	2.545	15.367	18.190	3.740	36.400	7.823	3.652	18.693	19.020	6.175	37.670
BRBZ	124.810	53.040	210.635	114.529	73.672	183.312	109.480	34.160	199.550	75.688	37.181	140.137	99.480	32.110	182.540
TTCP	48.160	20.850	98.690	34.572	24.770	65.876	48.650	24.995	91.830	35.811	25.470	59.272	51.865	23.840	103.020
MDMB	28.002	16.117	49.699	25.727	18.370	39.431	25.038	15.127	42.694	23.591	16.856	35.289	24.490	15.320	41.961
NDPA	189.522	114.323	297.187	220.818	167.607	283.814	204.797	119.218	304.237	221.415	161.474	290.342	189.094	115.814	293.958
Chemical	BBMD Reactome pathway			BMDExpress Reactome pathway			BBMD Individual gene			BMDExpress Individual pathway			KEGG pathway		
13 weeks															

TRBZ	7.490	2.140	34.900	6.952	3.560	15.299	11.070	2.900	34.885	5.757	3.019	13.708	9.175	2.760	28.260
BRBZ	105.870	43.910	184.870	82.220	56.968	139.323	113.060	49.300	206.510	91.211	61.671	148.874	117.640	45.250	252.100
TTCP	52.320	33.250	109.460	43.290	33.675	67.048	49.330	29.800	95.015	41.913	32.045	61.636	56.170	30.085	123.920
MDMB	29.560	13.838	58.719	22.711	15.335	37.591	28.760	14.444	58.345	23.481	16.925	40.054	28.486	13.573	58.308
NDPA	183.727	117.197	269.316	205.017	151.289	259.531	201.204	122.028	296.114	207.123	147.731	269.267	192.933	113.702	289.865

* BMD/BMDL/BMDU calculated by the model with the lowest AIC is chosen. These values calculated for NDPA (5 days, 2 weeks, 13 weeks), BRBZ (2 weeks, 13 weeks), TTCP (4 weeks, 13 weeks) and MDMB (2 weeks, 4 weeks and 13 weeks) are slightly different as the published values in Thomas et al. (2013b) due to the model updates.

## Footnotes

1 The BBMD user manual is available at: https://benchmarkdose.com/static/docs/BBMD_User_Manual.pdf.

## Abbreviations:

BBMD: Bayesian Benchmark Dose Modeling System
BMA: Bayesian model average
BEPOD: Biological Effect Point of Departure
BMD: Benchmark Dose
BMDL: Statistical Lower Bound of BMD
BMDS: Benchmark Dose Software
BMDU: Statistical Upper Bound of BMD
BMR: Benchmark Response
IVIVE: In-vitro to In-vivo Extrapolation
MCMC: Markov Chain Monte Carlo
NTP: National Toxicology Program
POD: Point of Departure

## References

[R1] Auerbach SS, Paules RS, 2018. Genomic dose response: Successes, challenges, and next steps. Current Opinion Toxicol. 11, 84–92.

[R2] Carpenter B, Gelman A, Hoffman MD, Lee D, Goodrich B, Betancourt M, Brubaker M, Guo J, Li P, Riddell A, 2017. Stan: A probabilistic programming language. J. Stat. Softw 76 (1) 10.18637/jss.v076.i01. PMC9788645 36568334

[R3] Clyde M, 2003. Model averaging. Subjective and objective Bayesian statistics.

[R28] Committee, E.S., Hardy A, Benford D, Halldorsson T., Jeger MJ, Knutsen KH, , 2017. Update: Use of the benchmark dose approach in risk assessment. EFSA J. 15, e04658. 32625254 10.2903/j.efsa.2017.4658 PMC7009819

[R4] Davis JA, Gift JS, Zhao QJ, 2011. Introduction to benchmark dose methods and us epa’s benchmark dose software (bmds) version 2.1. 1. Toxicol. Appl. Pharmacol 254 (2), 181–191. 21034758 10.1016/j.taap.2010.10.016

[R5] Ewald J, Soufan O, Xia J, Basu N, Cowen L, 2021. Fastbmd: An online tool for rapid benchmark dose–response analysis of transcriptomics data. Bioinformatics 37 (7), 1035–1036. 32761065 10.1093/bioinformatics/btaa700 PMC8128449

[R6] Fabregat A, Jupe S, Matthews L, Sidiropoulos K, Gillespie M, Garapati P, Haw R, Jassal B, Korninger F, May B, Milacic M, Roca CD, Rothfels K, Sevilla C, Shamovsky V, Shorser S, Varusai T, Viteri G, Weiser J, Wu G, Stein L, Hermjakob H, D’Eustachio P, 2018. The reactome pathway knowledgebase. Nucleic Acids Res. 46 (D1), D649–D655. 29145629 10.1093/nar/gkx1132 PMC5753187

[R7] Haber LT, Dourson ML, Allen BC, Hertzberg RC, Parker A, Vincent MJ, Maier A, Boobis AR, 2018. Benchmark dose (bmd) modeling: Current practice, issues, and challenges. Crit. Rev. Toxicol 48 (5), 387–415. 29516780 10.1080/10408444.2018.1430121

[R8] Igarashi Y, Nakatsu N, Yamashita T, Ono A, Ohno Y, Urushidani T, Yamada H, 2015. Open tg-gates: A large-scale toxicogenomics database. Nucleic Acids Res. 43 (D1), D921–D927. 25313160 10.1093/nar/gku955 PMC4384023

[R9] Johnson KJ, Auerbach SS, Costa E, 2020. A rat liver transcriptomic point of departure predicts a prospective liver or non-liver apical point of departure. Toxicol. Sci 176 (1), 86–102. 32384157 10.1093/toxsci/kfaa062 PMC7357187

[R10] Kanehisa M, Goto S, 2000. Kegg: Kyoto encyclopedia of genes and genomes. Nucleic Acids Res. 28, 27–30. 10592173 10.1093/nar/28.1.27 PMC102409

[R11] Klopfenstein DV, Zhang L, Pedersen BS, Ramírez F, Warwick Vesztrocy A, Naldi A, Mungall CJ, Yunes JM, Botvinnik O, Weigel M, Dampier W, Dessimoz C, Flick P, Tang H, 2018. Goatools: A python library for gene ontology analyses. Sci. Rep 8 (1) 10.1038/s41598-018-28948-z. PMC6052049 30022098

[R12] Mi H, Muruganujan A, Ebert D, Huang X, Thomas PD, 2019. Panther version 14: More genomes, a new panther go-slim and improvements in enrichment analysis tools. Nucleic Acids Res. 47, D419–D426. 30407594 10.1093/nar/gky1038 PMC6323939

[R13] National Research Council (NRC) (US) Committee on Applications of Toxicogenomic Technologies to Predictive Toxicology. Applications of Toxicogenomic Technologies to Predictive Toxicology and Risk Assessment. Washington (DC): National Academies Press (US); 2007. 20669432

[R14] Peddada S, Harris S, Zajd J, Harvey E, 2005. Oriogen: Order restricted inference for ordered gene expression data. Bioinformatics 21 (20), 3933–3934. 16109745 10.1093/bioinformatics/bti637

[R15] Phillips JR, Svoboda DL, Tandon A, Patel S, Sedykh A, Mav D, Kuo B, Yauk CL, Yang L, Thomas RS, Gift JS, Davis JA, Olszyk L, Merrick BA, Paules RS, Parham F, Saddler T, Shah RR, Auerbach SS, Valencia A, 2019. Bmdexpress 2: Enhanced transcriptomic dose-response analysis workflow. Bioinformatics 35 (10), 1780–1782. 30329029 10.1093/bioinformatics/bty878 PMC6513160

[R16] Program NT. 2018. Ntp research report on national toxicology program approach to genomic dose-response modeling. 30321009

[R17] Serra A, Saarimäki LA, Fratello M, Marwah VS, Greco D, Wren J, 2020. Bmdx: A graphical shiny application to perform benchmark dose analysis for transcriptomics data. Bioinformatics 36 (9), 2932–2933. 31950985 10.1093/bioinformatics/btaa030

[R18] Shao K, Gift JS, 2014. Model uncertainty and bayesian model averaged benchmark dose estimation for continuous data. Risk Anal. 34 (1), 101–120. 23758102 10.1111/risa.12078

[R19] Shao K, Shapiro AJ, 2018. A web-based system for bayesian benchmark dose estimation. Environ. Health Perspect. 126 (1), 017002. 10.1289/EHP1289. PMC6014690 29329100

[R20] Thomas RS, Clewell HJ, Allen BC, Wesselkamper SC, Wang NCY, Lambert JC, Hess-Wilson JK, Zhao QJ, Andersen ME, 2011. Application of transcriptional benchmark dose values in quantitative cancer and noncancer risk assessment. Toxicol. Sci 120 (1), 194–205. 21097997 10.1093/toxsci/kfq355

[R21] Thomas RS, Clewell HJ, Allen BC, Yang L, Healy E, Andersen ME, 2012. Integrating pathway-based transcriptomic data into quantitative chemical risk assessment: A five chemical case study. Mutation Research/Genetic Toxicology Environ. Mutagenesis 746 (2), 135–143. 10.1016/j.mrgentox.2012.01.007 22305970

[R22] Thomas RS, Himmelstein MW, Clewell III HJ, Yang Y, Healy E, Black MB, 2013a. Cross-species transcriptomic analysis of mouse and rat lung exposed to chloroprene. toxicological sciences 131:629–640. 23125180 10.1093/toxsci/kfs314

[R23] Thomas RS, Wesselkamper SC, Wang NCY, Zhao QJ, Petersen DD, Lambert JC, Cote I, Yang L, Healy E, Black MB, Clewell HJ, Allen BC, Andersen ME, 2013b. Temporal concordance between apical and transcriptional points of departure for chemical risk assessment. Toxicol. Sci 134 (1), 180–194. 23596260 10.1093/toxsci/kft094

[R24] User Manual and Technical Guidance for The Bayesian Benchmark Dose (BBMD) Analysis System Version 2.0 (2021.8.11) (https://benchmarkdose.com/static/docs/BBMD_User_Manual.pdf).

[R25] Williams D 1971. A test for differences between treatment means when several dose levels are compared with a zero dose control. Biometrics: 103–117. 5547548

[R26] Williams DA, 1972. The comparison of several dose levels with a zero dose control. Biometrics 519–531. 10.2307/2556164. 5037867

[R27] Yang L, Allen BC, Thomas RS, 2007. Bmdexpress: A software tool for the benchmark dose analyses of genomic data. BMC Genomics 8, 1–8. 17961223 10.1186/1471-2164-8-387 PMC2198920

