# Supplementary material for: A computational system for Bayesian benchmark dose estimation of genomic data in BBMD
Source: Environ Int. Author manuscript; Available in PMC 2022 Mar 19. (PMC8934139; doi:10.1016/j.envint.2022.107135)
Supplement: 1 [file NIHMS1778866-supplement-1.docx]

Supplemental Material

**A Computational System for Bayesian Benchmark Dose Estimation of Genomic Data in BBMD**

Chao Ji ^1^, Andrew Weissmann^2^, Kan Shao^1,*^

1. Department of Environmental and Occupational Health, School of Public Health, Indiana University – Bloomington, Bloomington, IN 47405

2. DREAM Tech, LLC, Bloomington, IN 47401

* Corresponding Author:

Kan Shao, Ph.D.

Associate Professor

Department of Environmental and Occupational Health

Indiana University School of Public Health

1025 E. Seventh Street

Bloomington, IN 47405

Tel: 812-856-2725

Fax: 812-856-2488

Email: [kshao@indiana.edu](mailto:kshao@indiana.edu)

1. **Workflow of the BBMD system**

The workflow of the BBMD system is in Figure S1. Step by step description of the BBMD system can be found through the BBMD user manual (<https://benchmarkdose.com/static/docs/BBMD_User_Manual.pdf>).


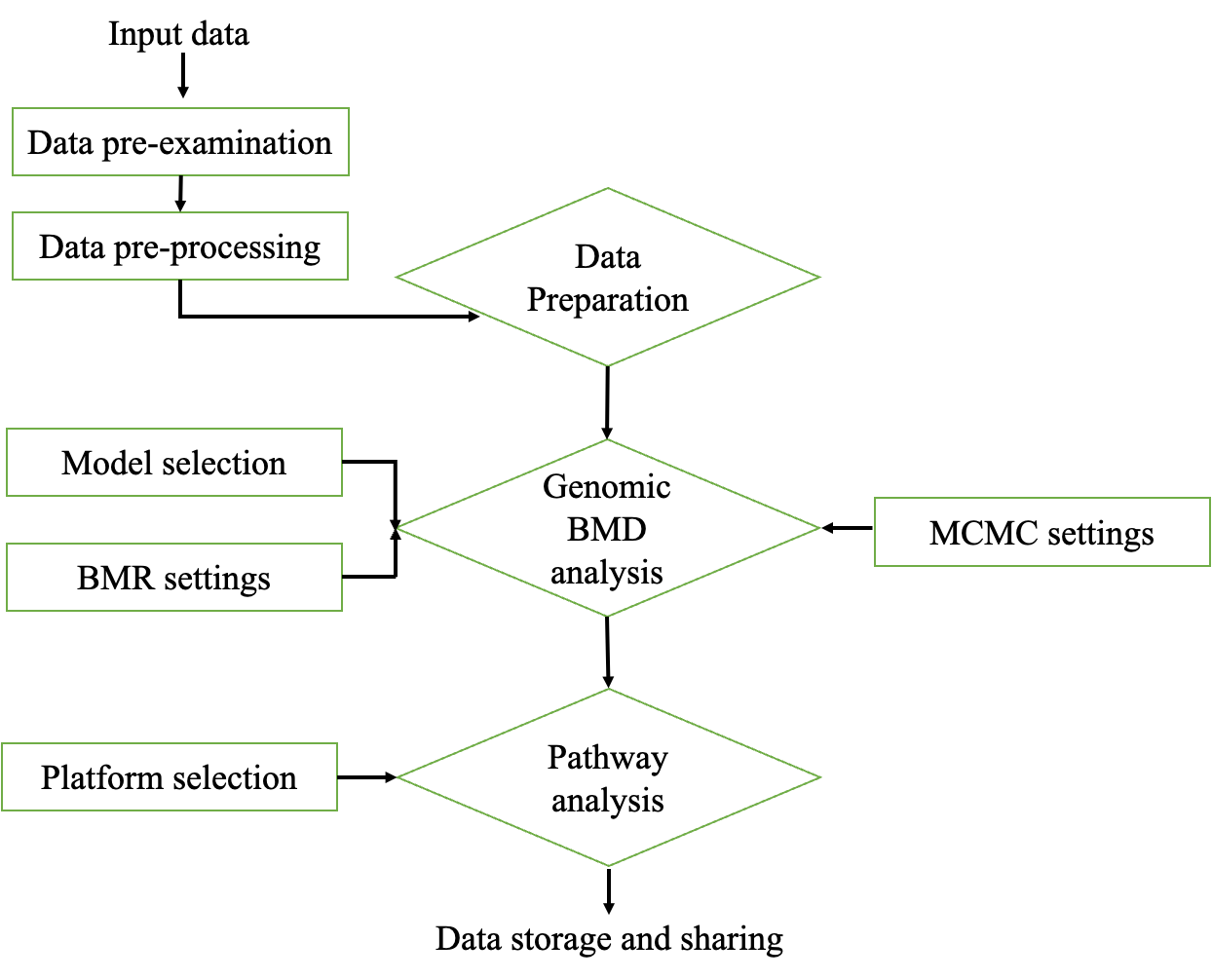


Figure S1 A flowchart for genomic BMD analysis in the BBMD system

1. **Available dose response models in the BBMD system**

The available dose-response models in the BBMD system include models below.

1. Linear Model:

$f\left( dose \right)=a+b\times dose, a>0$, (S1)

$$a\sim Uniform\left( 0, a_{upper} \right); b\sim Uniform\left( b_{lower}, b_{upper} \right)$$

1. Power Model:

$f\left( dose \right)=a+b\times{dose}^{g}$, (S2)

$$a\sim Uniform\left( 0, a_{upper} \right); b\sim Uniform\left( b_{lower}, b_{upper} \right); g\sim Uniform\left( 1, 15 \right)$$

$$a>0, g\geq1$$

1. Hill Model:

$f\left( dose \right)=a+\frac{b\times{dose}^{g}}{c^{g}+{dose}^{g}}$, (S3)

$$a\sim Uniform\left( 0, a_{upper} \right); b\sim Uniform\left( b_{lower}, b_{upper} \right); c\sim Uniform\left( 0, 15 \right); g\sim Uniform\left( 1, 15 \right)$$

$$a>0, c>0, g\geq1$$

1. Exponential 2 Model:

$f\left( dose \right)=a\times e^{b\times dose}, a>0$, (S4)

$a\sim Uniform\left( 0, a_{upper} \right); b\sim Uniform\left( 0, 50 \right)$ for increasing trend or $Uniform\left( -50, 0 \right)$ for decreasing trend.

1. Exponential 3 Model:

$\left( dose \right)=a\times e^{b\times{dose}^{g}}$ (S5)

$$a>0,g\geq1$$

$$a\sim Uniform\left( 0, a_{upper} \right);$$

$b\sim Uniform\left( 0, 50 \right)$ for increasing trend or $Uniform\left( -50, 0 \right)$ for decreasing trend;

$$g\sim Uniform\left( 1, 15 \right);$$

1. Exponential 4 Model:

$f\left( dose \right)=a\times\left( c-\left( c-1 \right)\times e^{-b\times dose} \right)$ (S6)

$$a>0,b>0,c>0$$

$a\sim Uniform\left( 0, a_{upper} \right); b\sim Uniform\left( 0, 100 \right);$ $c\sim Uniform\left( 0, 1 \right)$ for decreasing trend or $c\sim Uniform\left( 1, 15 \right)$ for increasing trend.

1. Exponential 5 Model:

$f\left( dose \right)=a\times\left( c-\left( c-1 \right)\times e^{-(b\times d{ose)}^{g}} \right)$ (S7)

$$a>0,b>0,c>0, g\geq1$$

$a\sim Uniform\left( 0, a_{upper} \right); b\sim Uniform\left( 0, 100 \right);$ $c\sim Uniform\left( 0, 15 \right)$ for decreasing trend or

$c\sim Uniform\left( 1, 15 \right)$ for increasing trend; $g\sim Uniform\left( 1, 15 \right)$.

1. **Datasets**
   1. Transcriptional data

The transcriptional data for model comparisons between BBMD and BMDExpress are summarized in Table S.1. Six chemicals (TRBZ, BRBZ, TTCP, MDMB, NDPA and HZBZ) were dosed orally to rat. The transcriptional changes of each chemical using microarrays were measured for 5 days, 2, 4, and 13 weeks. Datasets are downloaded from <https://www.ncbi.nlm.nih.gov/geo/query/acc.cgi?acc=GSE45892>.

Table S.1 A summary of dose-response microarray data (GSE45892) for model comparisons

| Chemical | Abbreviation | Doses | Target organs |
| --- | --- | --- | --- |
| 1,2,4-Tribromobenzene | TRBZ | 0, 2.5, 5, 10, 25, 75 mg/kg | Liver |
| Bromobenzene | BRBZ | 0, 25, 100, 200, 300, 400 mg/kg | Liver |
| 2,3,4,6-Tetrachlorophenol | TTCP | 0, 10, 25, 50, 100, and 200 mg/kg | Liver |
| 4,4’-Methylenebis (N, N-dimethyl) benzenamine | MDMB | 0, 50, 200, 375, 500, 750 ppm | Thyroid |
| N-Nitrosodiphenylamine | NDPA | 0, 250, 1000, 2000, 3000, 4000 ppm | Bladder |
| Hydrazobenzene | HZBZ | 0, 5, 20, 80, 200, 300 ppm | Liver |

- 1. Observational data

To find the correlation relationship between transcriptional data and observational data, in (Thomas et al. 2013), the target organs of each chemical were also analyzed for traditional histological and organ weight changes. The dataset for tumor incidences in the original cancer bioassays that were used in our manuscript are summarized in Table S.3. Dichotomous data in Table S.3 are obtained from supplementary data (Thomas et al. 2013) toxsci_13_0077_File003 and continuous data in Table S.3 are obtained from supplementary data toxsci_13_0077_File004. These datasets are used to calculate the BMDL-BMD-BMDU values in Table 3.

The transcriptional data for BEPOD and POD correlation analysis include five chemicals TRBZ, BRBZ, TTCP, MDMB and NDPA in Table S.1 and the 64 molecules from the Open TG-Gates database (<https://dbarchive.biosciencedbc.jp/en/open-tggates/download.html>). The 64 chemicals are selected as they have in-vivo and in-vitro transcriptional data as well as the apical data. These 64 chemicals can be found in the supplement Table S.2 file. As a note, the fold change of genomic expressions of chemicals amiodarone and azathioprine at 3h, 6h, 9h and 24h are less than 2 and *no* probe pass the preprocessing. Same for the dose response data of chemical moxisylyte at 4-day. As a result, 62 chemicals at 3h, 6h, 9h and 24h, 63 chemicals at 4-day and 64 chemicals at 8-day, 15 day, and 29 days are available for the correlation analysis. The data plotted in Figure 6 are summarized in Table S.2. In Table S.2, the BMD values are derived from the pathway analysis in BBMD. The apical BMDs for five chemicals (TRBZ, BRBZ, TTCP, MDMB and NDPA) and the cancer apical BMDs of MDMB and NDPA are obtained from (Thomas et al. 2013b). The apical BMDs of the 64 chemicals from the Open TG-Gates database are obtained from (Johnson et al. 2020).

Table S.3 A summary of observational data for transcriptional data and observational data correlation analysis

| Chemical | Endpoint | Type of Data |
| --- | --- | --- |
| 5 Days |  |  |
| TRBZ | Absolute liver weight | Continuous individual |
| BRBZ | Centrilobular Multifocal Inflammation (subacute) | Dichotomous |
| TTCP | Absolute liver weight | Continuous individual |
| MDMB | Follicular Cell Hypertrophy | Dichotomous |
| NDPA | Absolute liver weight | Continuous individual |
| HZBZ | Absolute liver weight | Continuous individual |
| 2 Weeks |  |  |
| TRBZ | Centrilobular Hepatocytic Hypertrophy | Dichotomous |
| BRBZ | Absolute liver weight | Continuous individual |
| TTCP | Absolute liver weight | Continuous individual |
| MDMB | Follicular Cell Hypertrophy | Dichotomous |
| NDPA | Diffuse Transitional Epithelial Hyperplasia | Dichotomous |
| HZBZ | Absolute liver weight | Continuous individual |
| 4 Weeks |  |  |
| TRBZ | Centrilobular Hepatocytic Hypertrophy | Dichotomous |
| BRBZ | Absolute liver weight | Continuous individual |
| TTCP | Centrilobular Hepatocytic Vacuolation | Dichotomous |
| MDMB | Follicular Cell Hypertrophy | Dichotomous |
| NDPA | Diffuse Transitional Epithelial Hyperplasia | Dichotomous |
| HZBZ | Absolute liver weight | Continuous individual |
| 13 Weeks |  |  |
| TRBZ | Absolute liver weight | Continuous individual |
| BRBZ | Absolute liver weight | Continuous individual |
| TTCP | Centrilobular Hepatocytic Vacuolation | Dichotomous |
| MDMB | Follicular Cell Hypertrophy | Dichotomous |
| NDPA | Diffuse Transitional Epithelial Hyperplasia | Dichotomous |
| HZBZ | Absolute liver weight | Continuous individual |

1. **Modeling setting**
   1. Transcriptional data

Prior to the BMD analysis, one-way ANOVA method is used to identify the statistically significant gene sets. P-value 0.05 and fold change 2 are applied. These filtered data (Table S.1) are input for BMD analysis, standard models in BBMD (Linear, Power, Hill, Exp2, Exp3, Exp4 and Exp5) were fit to the dose-response data of each probe set. The default parameters for the BMD and pathway analysis are summarized below.

- BMDExpress
- BMD settings
- Maximum iteration: 250
- Confidence Level: 0.95
- Constant Variance: True
- BMR Type: Standard Deviation
- BMR Factor: 1.0
- Restrict Power: >=1
- Best Model Selection: Nested Chi Square to select best poly model followed by lowest AIC
- Nested Chi Square p-value cutoff: 0.05
- Fit Selected Models with Multiple Threads: 100
- Number of Available Processors on Machine: 4
- BMDL and BMDU Model Selection: Compute and utilize in best model selection
- Flag Hill Model with ‘k’ Parameter <: 1/3 of Lowest Positive Dose
- Best Model Selection with Flagged Hill Model: Select Next Best Model with P-Value >0.05
- Category Analysis settings
- Remove Promiscuous Probe: True
- Remove BMD > Highest Dose from Category Descriptive Statistics: True
- Identify conflicting probe sets: 0.5
- BBMD
- BMD settings
- BMR Type: SD Change
- BMR Value: 1
- MCMC settings
- Iterations: 30000
- Number of chains: 1
- Warmup percent: 50%
- Seed: 11696
- Pathway Platform: GPL 16985
  1. Observational data

In (Thomas et al. 2013), the tumor incidences data (Table S.3) were analyzed using the BMD models in BMDS to identify noncancer points-of-departure. Their study has BMR as 0.1 multiply relative change. As the BMDS model has been updated, we rerun the BMDS 3.2 system to get the updated noncancer points-of-departure. For the results of BMDS 3.2, model with lowest AIC is chosen as ‘best model’. Meanwhile, the same datasets input BBMD for a comparison, and all standard models in the BBMD system are used for BMD analysis. The default parameters for the BMD analysis are summarized below.

- BMDS 3.2
  - Dichotomous data
    - Select Model Type: Dichotomous
    - Models: MLE (frequentist restricted) includes Dichotomous Hill, Gamma, Log-logistic, Log-Probit, Multistage, and Weibull
    - Risk Type: extra risk
    - BMR: 0.1
    - Confidence Level: 0.95
    - Background: Estimated
  - Continuous data
    - Select Model Type: Continuous
    - Models: MLE (frequentist restricted) includes Exponential, Hill, Polynomial, and Power
    - Risk Type: Rel. Dev.
    - BMRF: 0.1
    - Confidence Level: 0.95
    - Polynomial Restriction: Use dataset adverse direction
    - Distribution: Normal
    - Variance: Constant
- BBMD
- BMD settings
- BMR Type: relative change
- BMR Value: 0.1
- MCMC settings
- Iterations: 30000
- Number of chains: 1
- Warmup percent: 50%
- Seed: 21981

1. **BMD analysis comparisons results**

5.1 Single model comparisons

BMD-BMD and BMDL-BMDL plots comparisons are in Figures S2-S19.

5.2 Comparison of BEPOD to Apical POD

The range plots of BEPOD to apical POD are in Figures S20-S24. The three dots from left to right in Figures S20-S24 represent BMDL, BMD, and BMDU.

The abbreviations of y axis in Figures S20-S24 are explained below.

- BMDS_POD: the BMDL-BMD-BMDU values calculated by BMDS 3.2
- BBMD_POD: the BMDL-BMD-BMDU values calculated by the ‘BMD analysis for Single Dataset’ module of BBMD
- KEGG: the BMDL-BMD-BMDU values of KEGG pathway analysis calculated by BBMD
- BMDE_Rea: the BMDL-BMD-BMDU values of REACTOME pathway analysis calculated by BMDExpress 2.0
- BBMD_Rea: the BMDL-BMD-BMDU values of REACTOME pathway analysis calculated by BBMD
- BMDE_ind: the BMDL-BMD-BMDU values of individual pathway analysis calculated by BMDExpress 2.0
- BBMD_ind: the BMDL-BMD-BMDU values of individual pathway analysis calculated by BBMD
- BMDE_GO: the BMDL-BMD-BMDU values of GO pathway analysis calculated by BMDExpress 2.0
- BBMD_GO: the BMDL-BMD-BMDU values of GO pathway analysis calculated by BBMD

5.3 Cumulative Distribution Comparison of Pathway Analysis on Log Scale

To better visualize the differences of sensitive pathways, we take the log scale of both y-axis and x-axis. The cumulative distributions of the BMD median of the 24 datasets using individual gene analysis, GO analysis, and REACTOME pathway analysis are shown in Figures S24-S26.


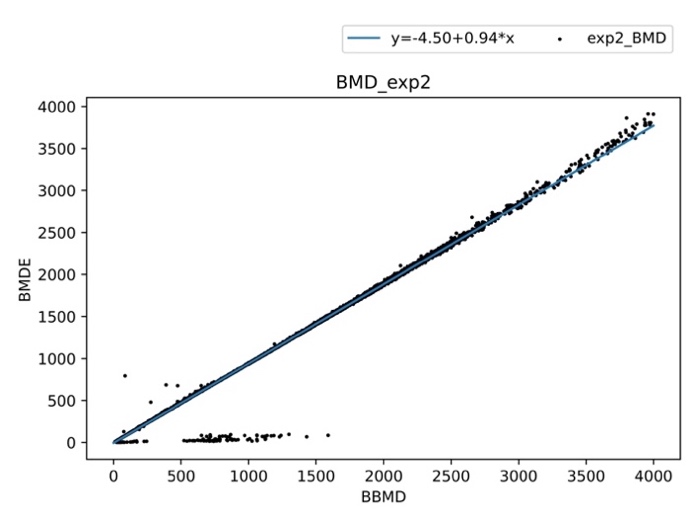

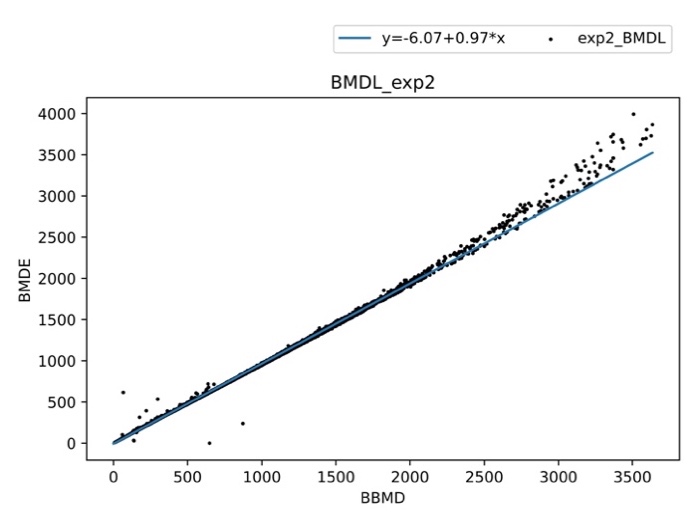


Figure S2 The BMD-BMD plot for the Exponential 2 model Figure S3 The BMDL-BMDL plot for the Exponential 2 model


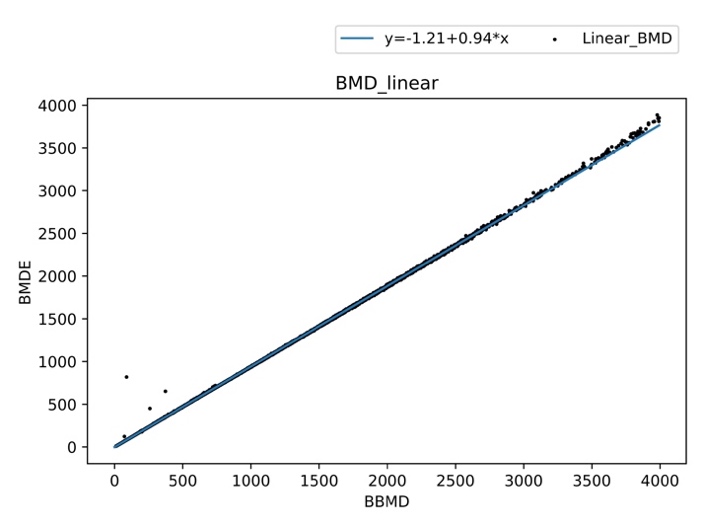

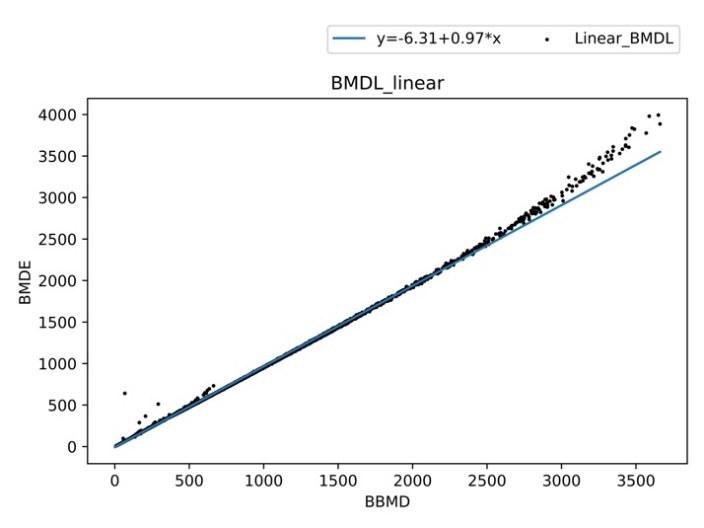


Figure S4 The BMD-BMD plot for the Linear model Figure S5 The BMDL-BMDL plot for the Linear model


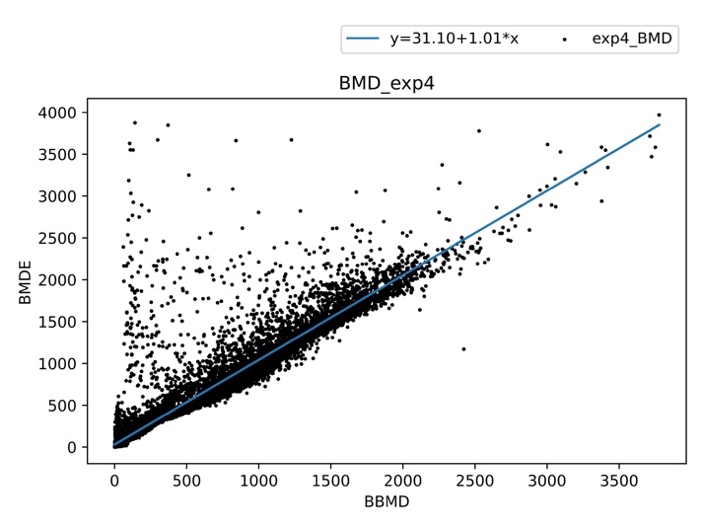

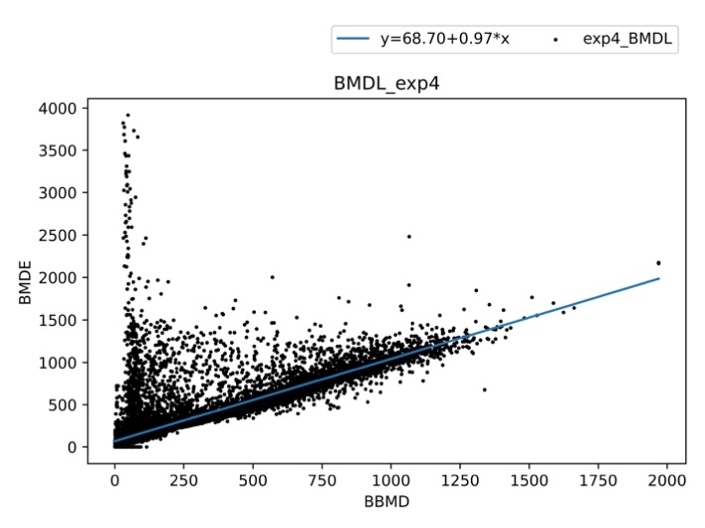


Figure S6 The BMD-BMD plot for the Exponential 4 model Figure S7 The BMDL-BMDL plot for the Exponential 4 model


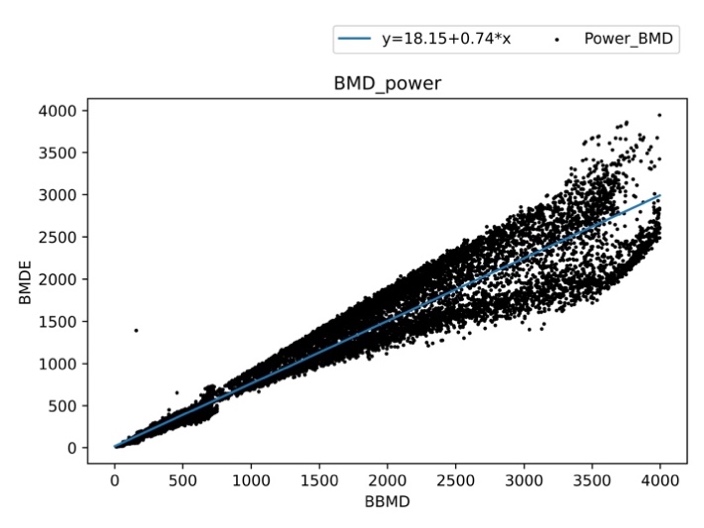

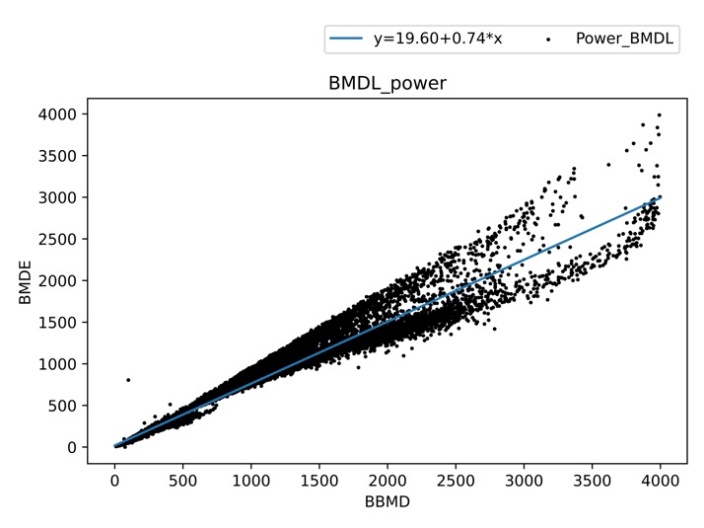


Figure S8 The BMD-BMD plot for the Power model Figure S9 The BMDL-BMDL plot for the Power model


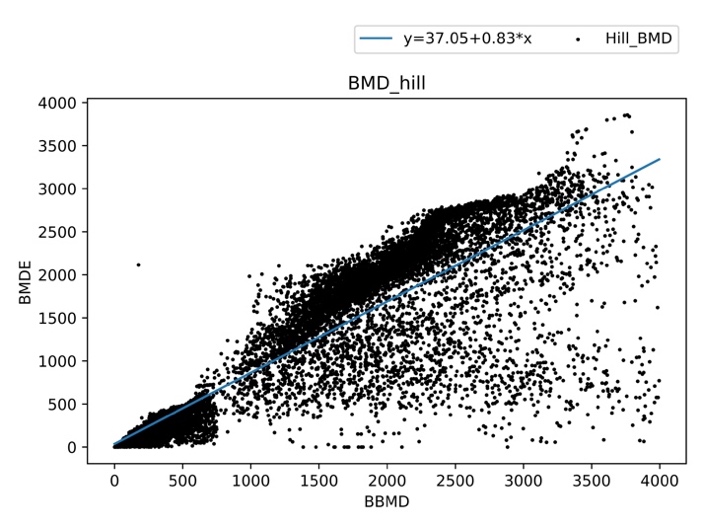

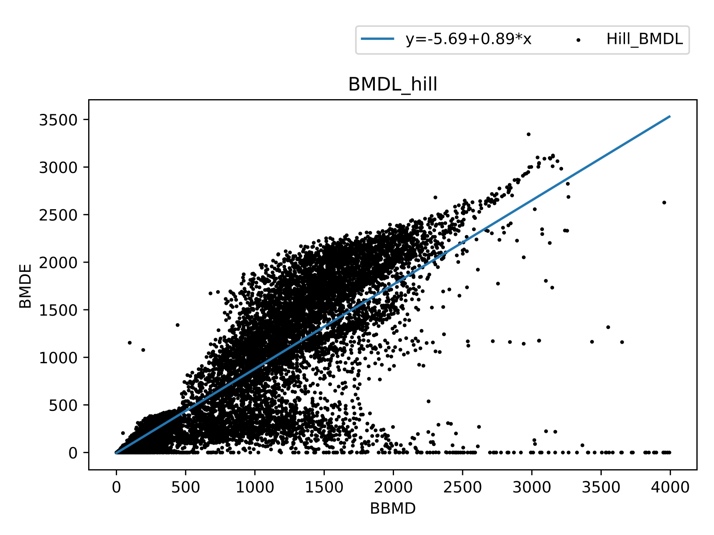


Figure S10 The BMD-BMD plot for the Hill model Figure S11 The BMDL-BMDL plot for the Hill model


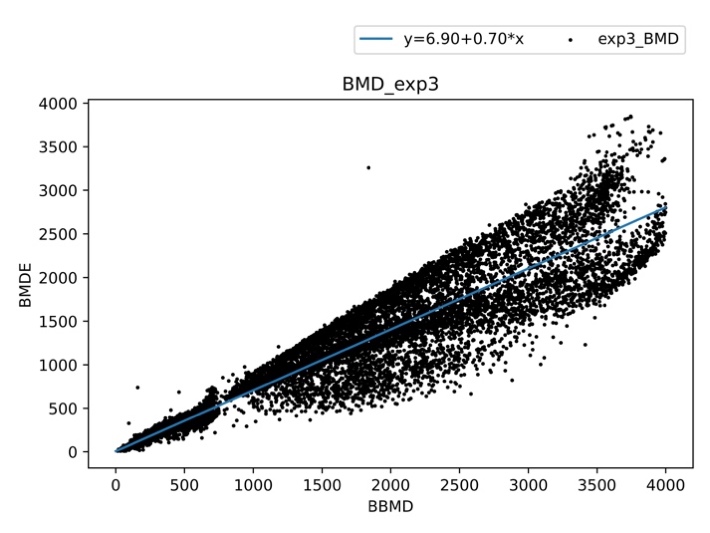

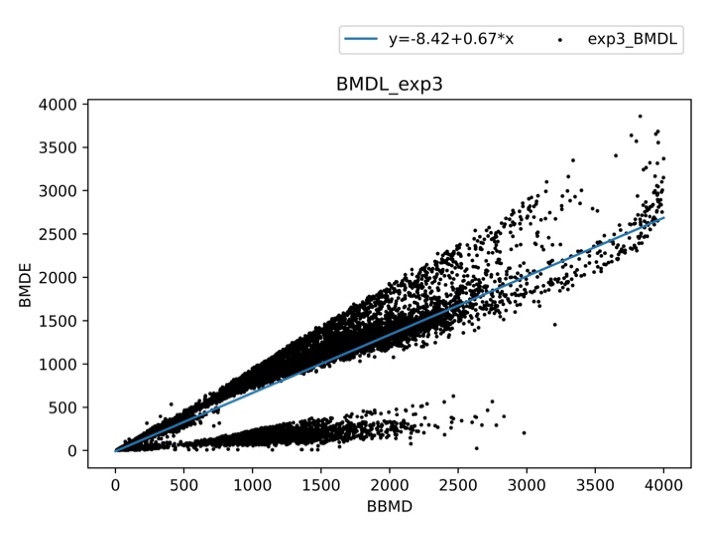


Figure S12 The BMD-BMD plot for the Exponential 3 model Figure S13 The BMDL-BMDL plot for the Exponential 3 model


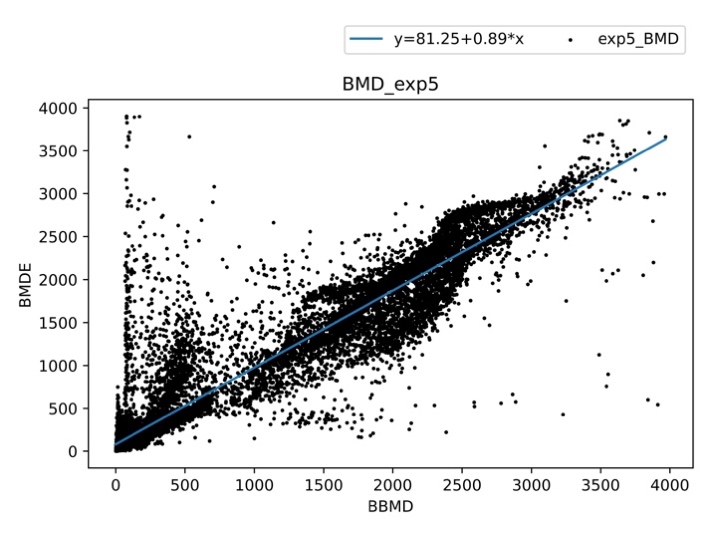

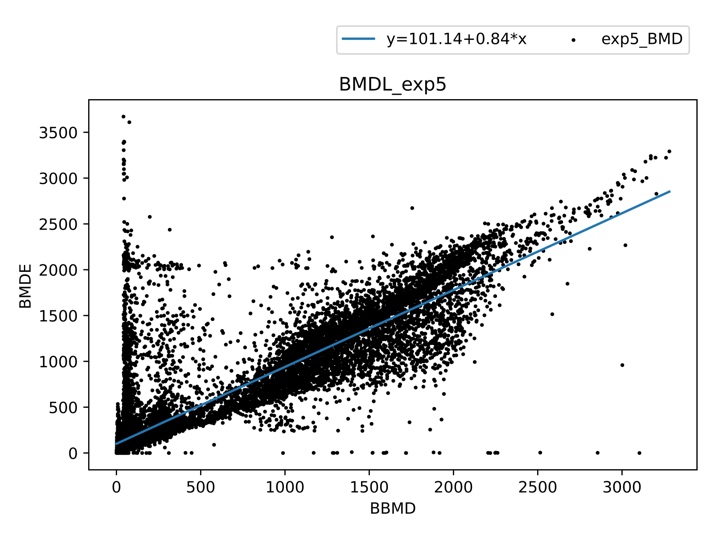


Figure S14 The BMD-BMD plot for the Exponential 5 model Figure S15 The BMDL-BMDL plot for the Exponential 5 model


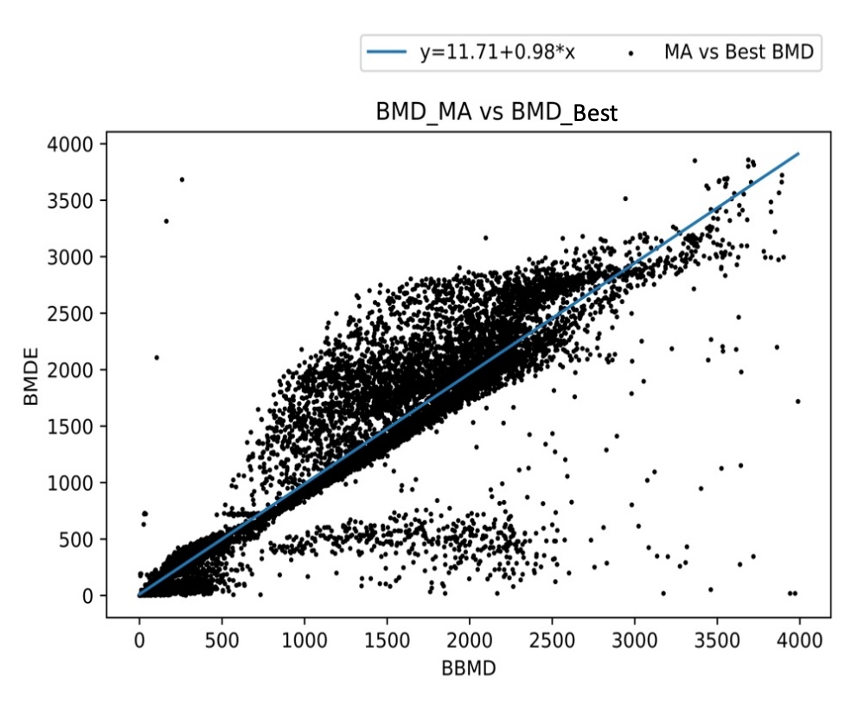

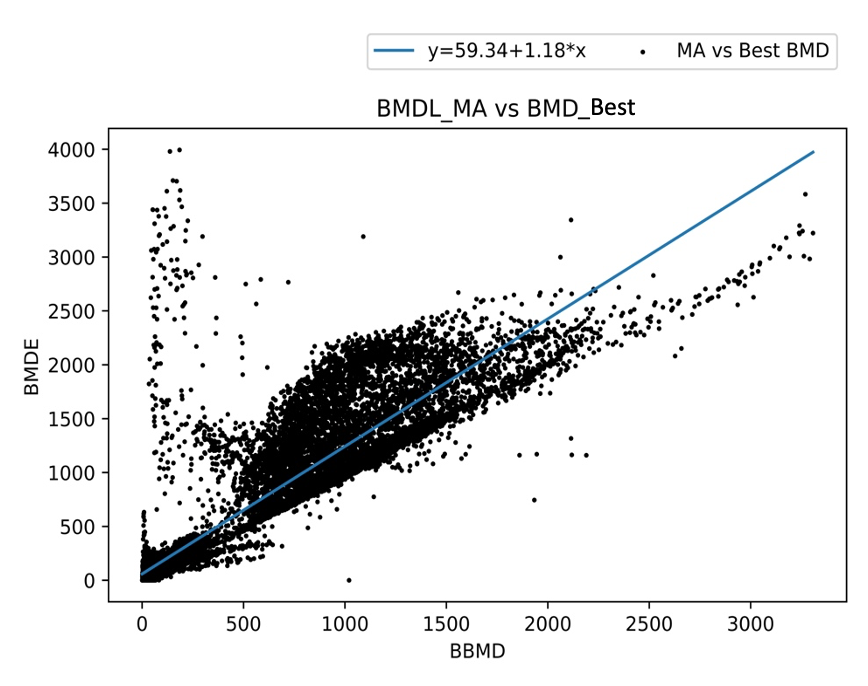


Figure S16 The BMD-BMD plot for the BMA estimates and Figure S17 The BMDL-BMDL plot for the BMA estimates and ‘best’ model removing BMD estimates>max dose ‘best’ model removing BMD estimates>max dose


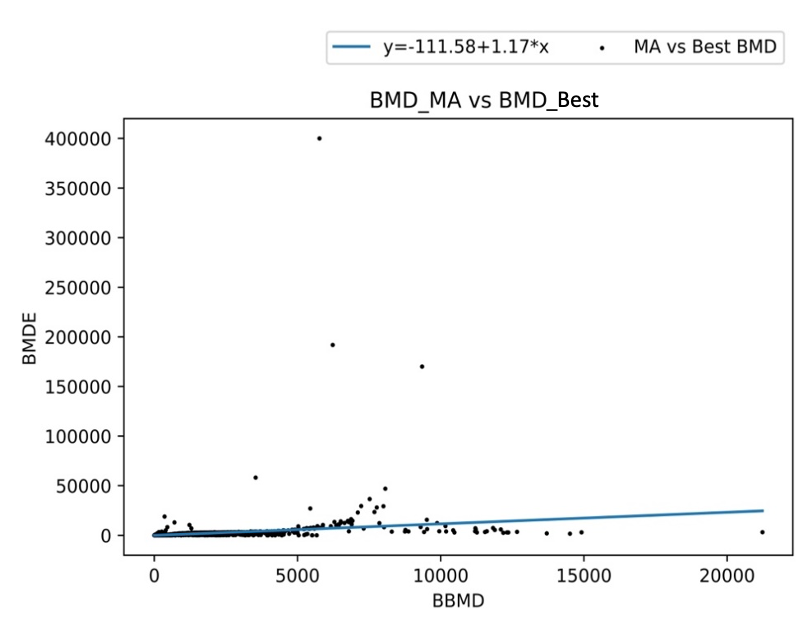

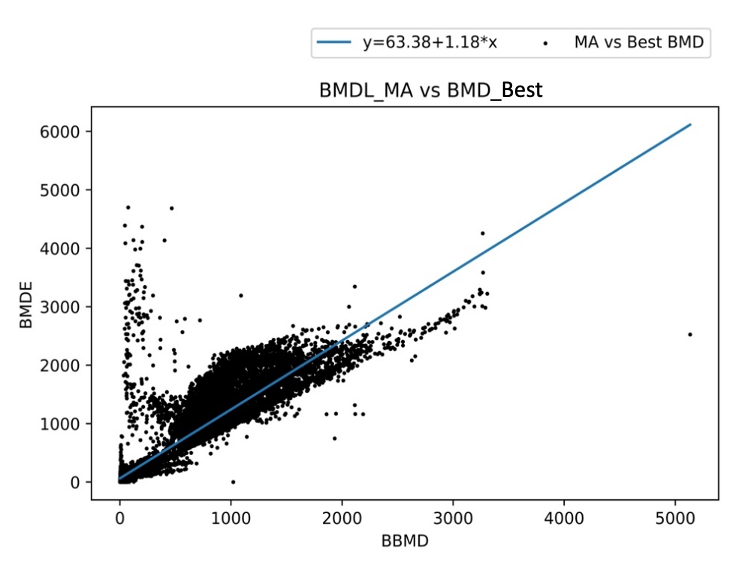
*Figure S18 The BMD-BMD plot for the BMA estimates and Figure S19 The BMDL-BMDL plot for the BMA estimates and ‘best’ model ‘best’ model*

- BRBZ

(a) (b)


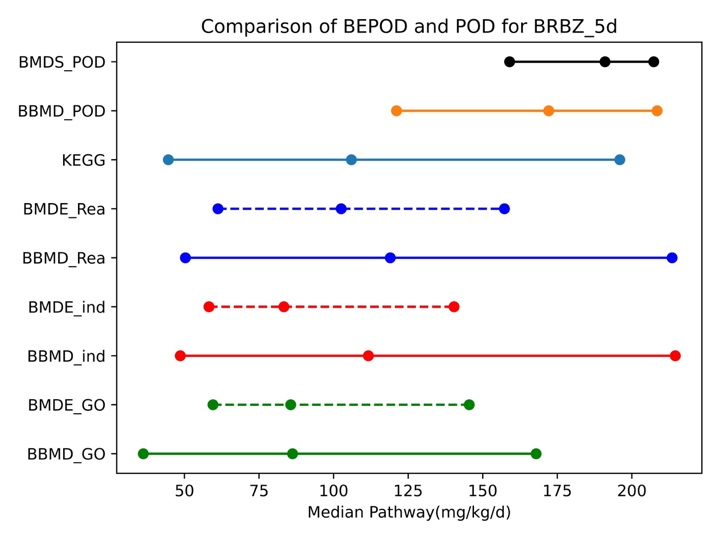

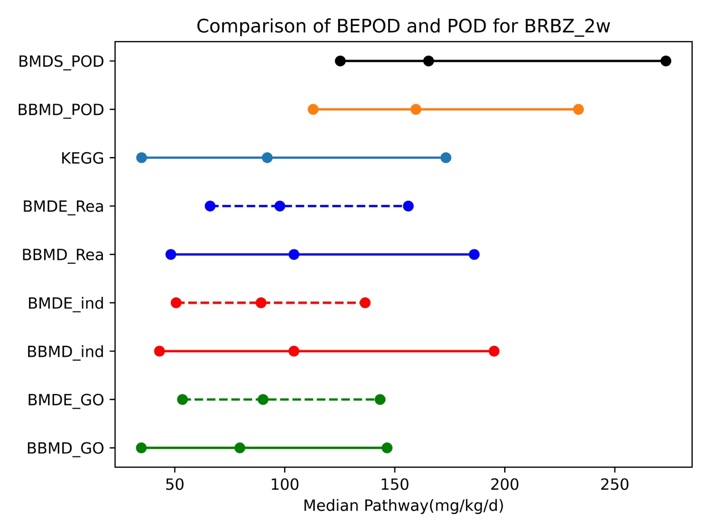


(c) (d)


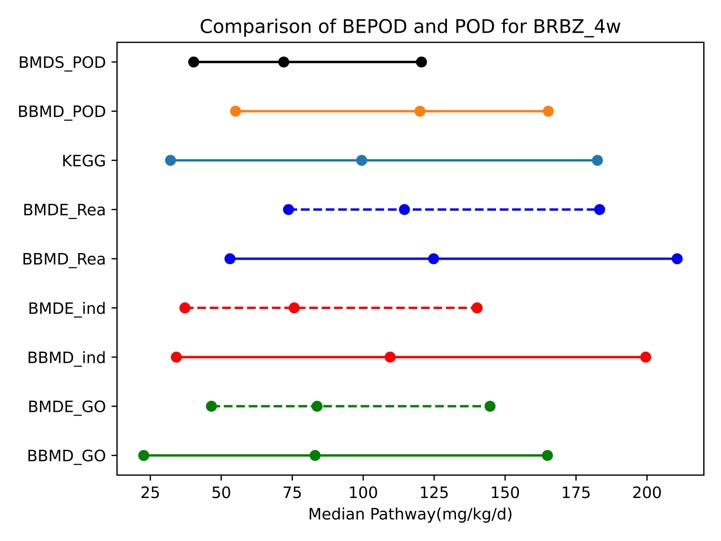

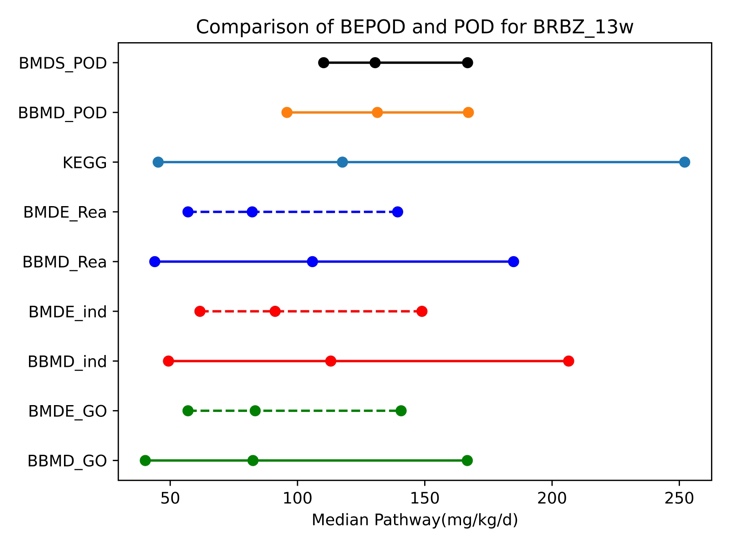


Figure S20 Comparison between BEPOD and apical POD for BRBZ at (a) 5 days, (b) 2 weeks, (c) 4 weeks, and (d) 13 weeks

- MDMB

(a) (b)


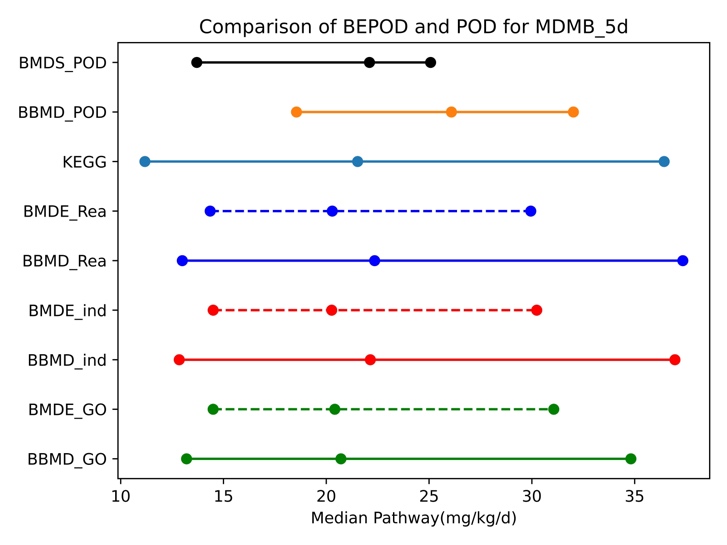

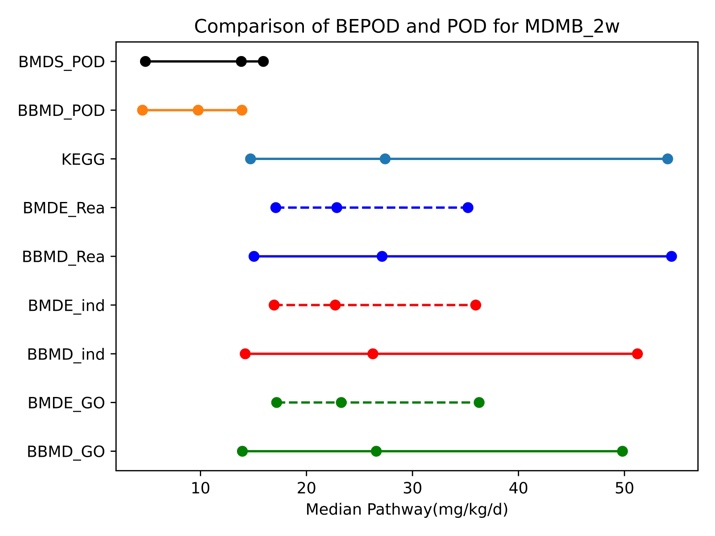


(c) (d)


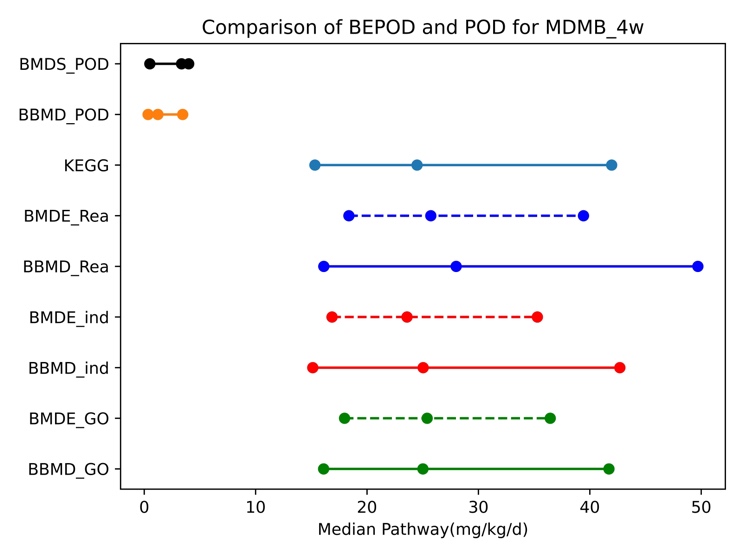

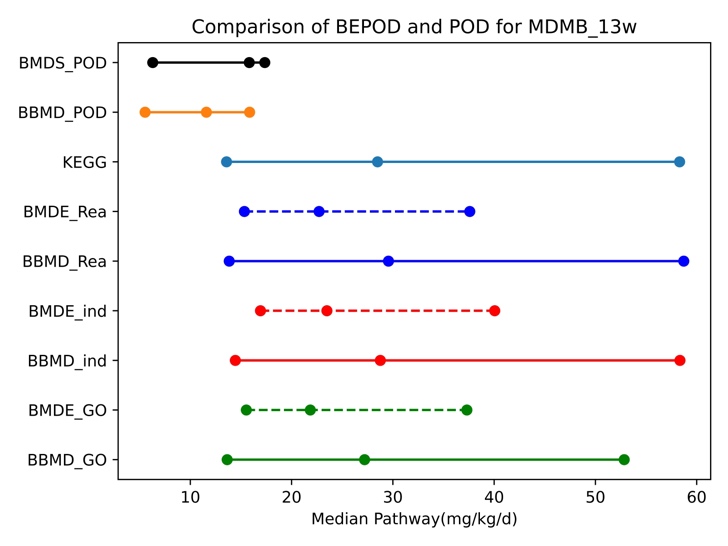


Figure S21 Comparison between BEPOD and apical POD for MDMB at (a) 5 days, (b) 2 weeks, (c) 4 weeks, and (d) 13 weeks

- NDPA

(a) (b)


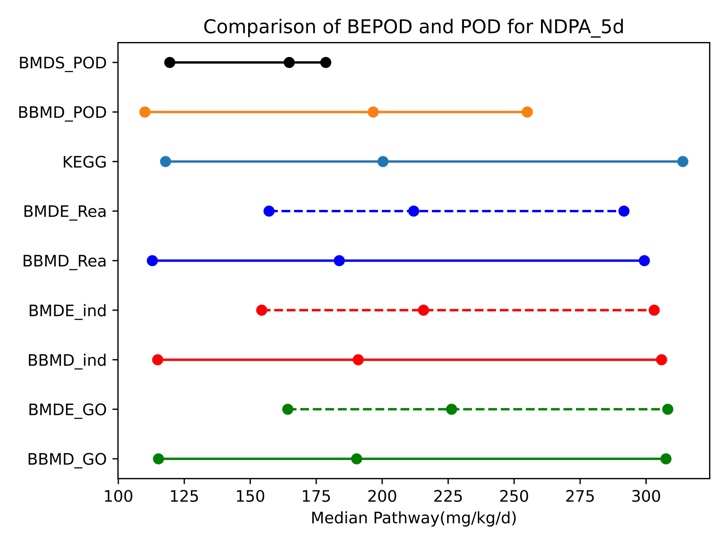

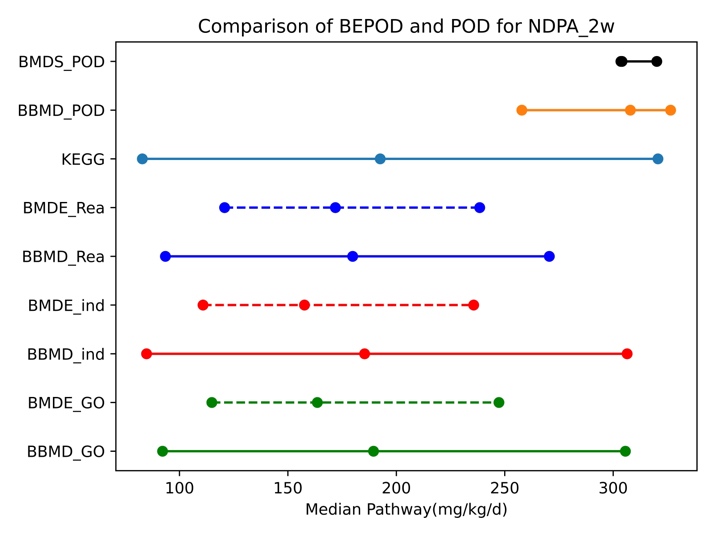


(c) (d)


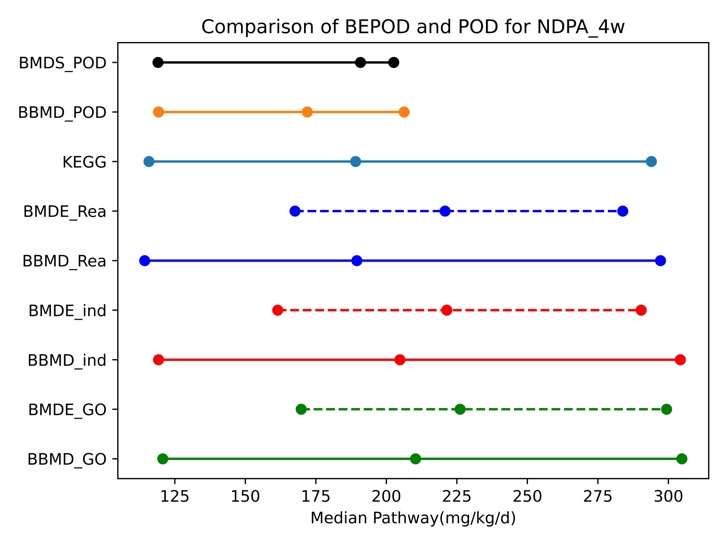

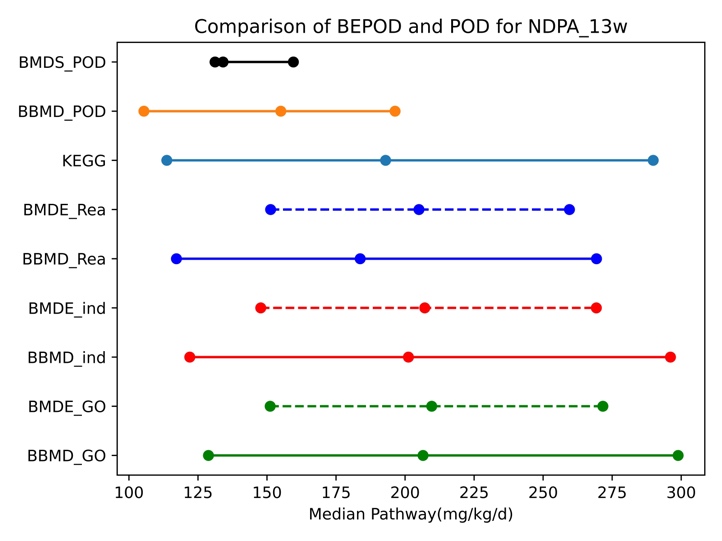


Figure S22 Comparison between BEPOD and apical POD for NDPA at (a) 5 days, (b) 2 weeks, (c) 4 weeks, and (d) 13 weeks

- TRBZ

(a) (b)


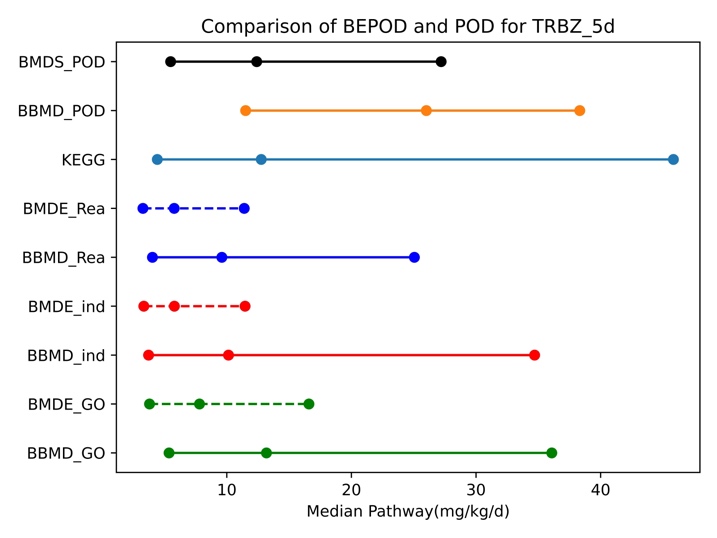

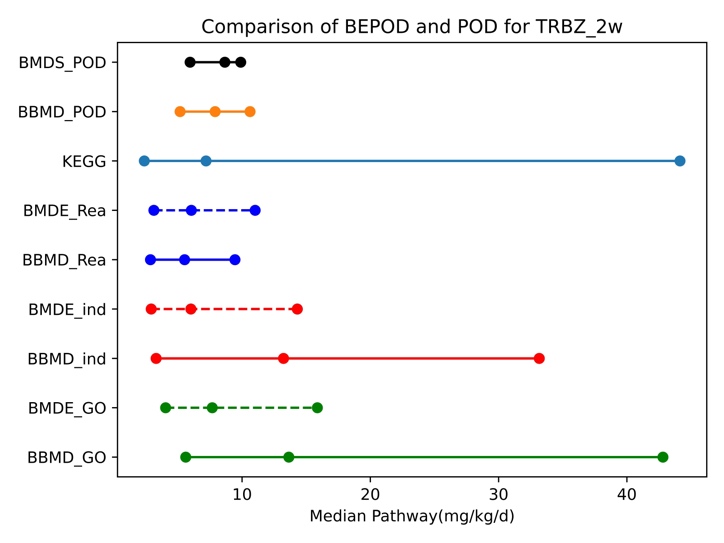


(c) (d)


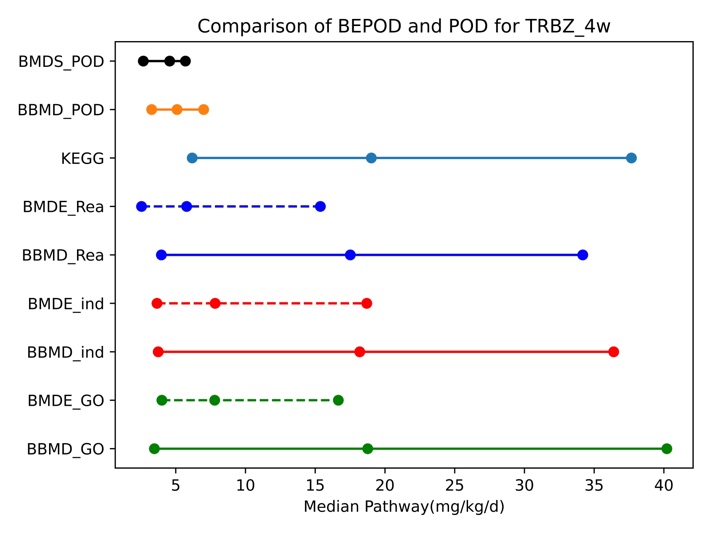

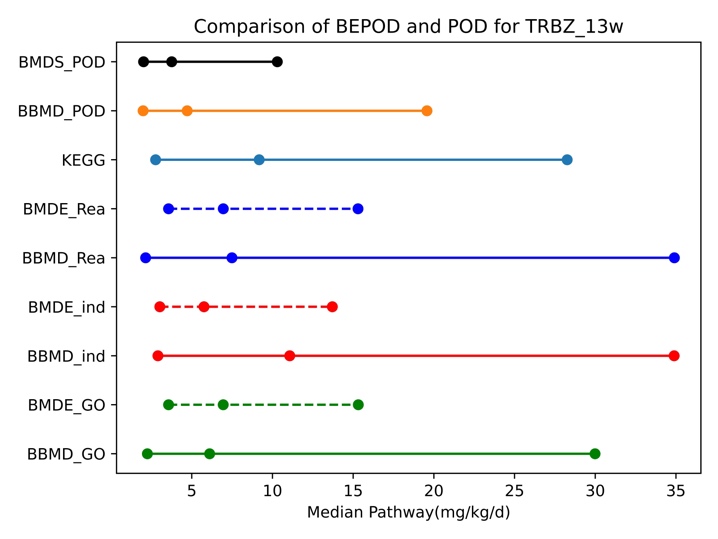


Figure S23 Comparison between BEPOD and apical POD for TRBZ at (a) 5 days, (b) 2 weeks, (c) 4 weeks, and (d) 13 weeks

- TTCP

(a) (b)


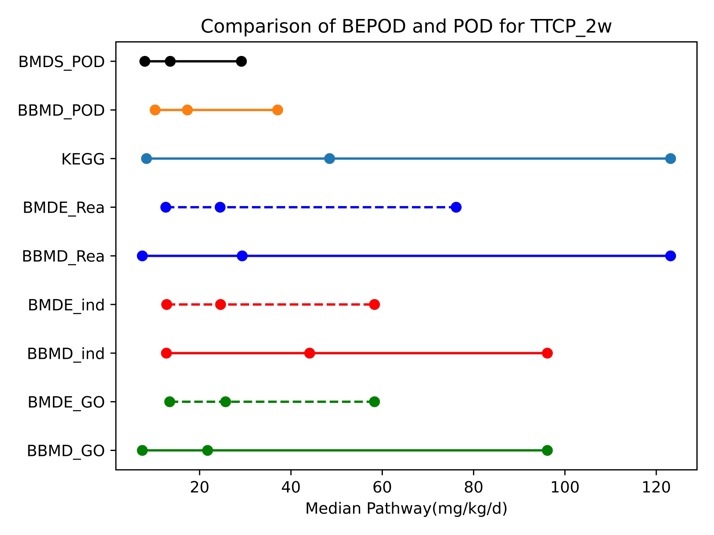

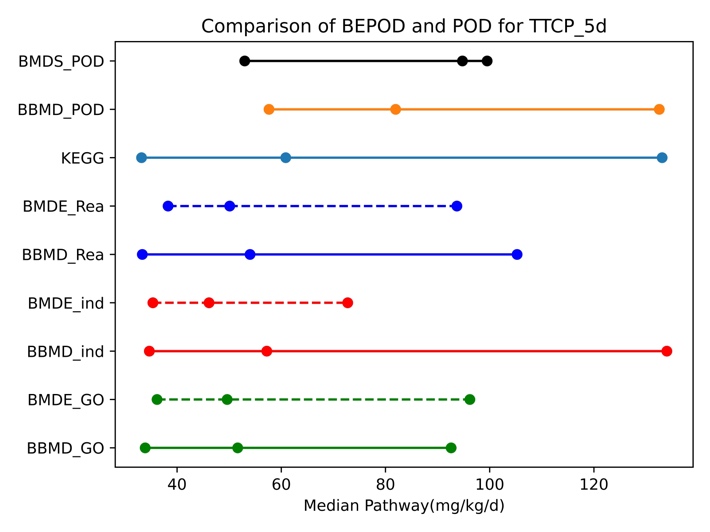


(c) (d)


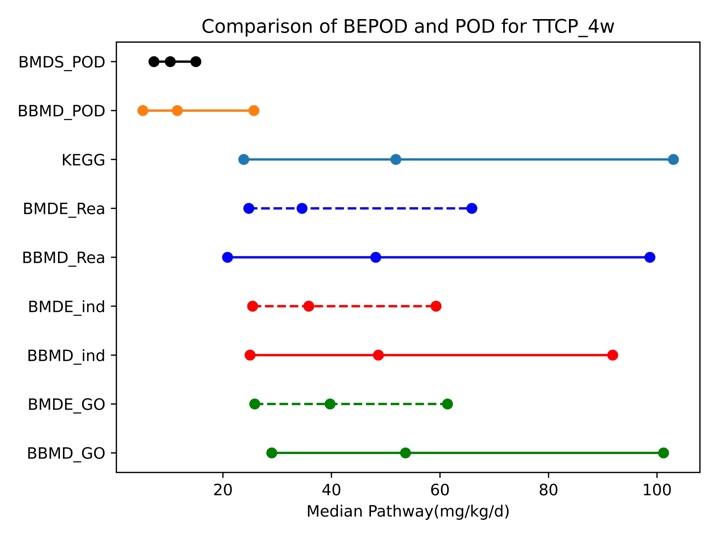

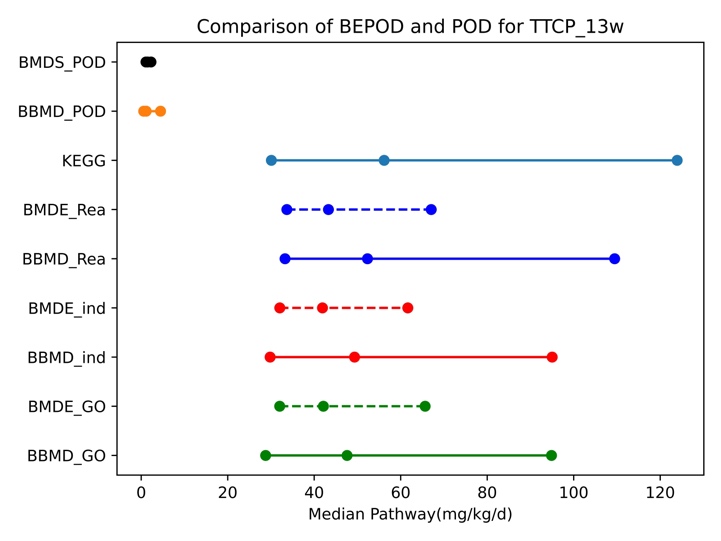


Figure S24 Comparison between BEPOD and apical POD for TRBZ at (a) 5 days, (b) 2 weeks, (c) 4 weeks, and (d) 13 week

- Individual gene analysis comparison

(a) (b)


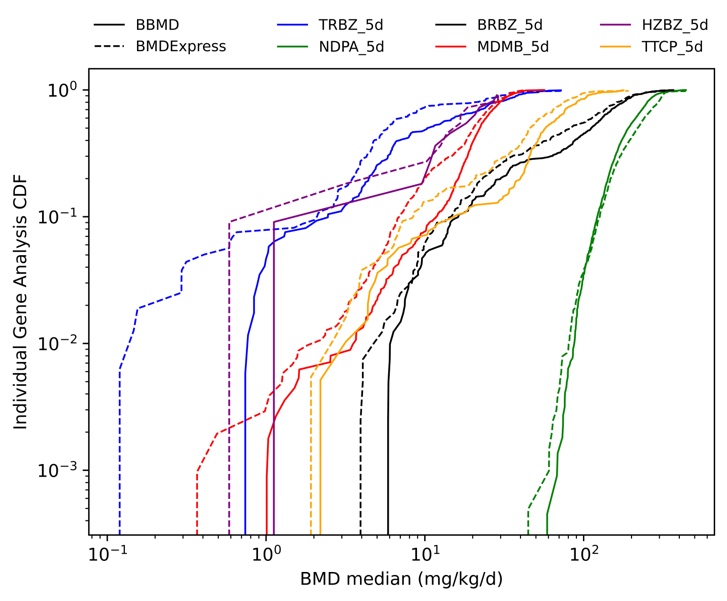

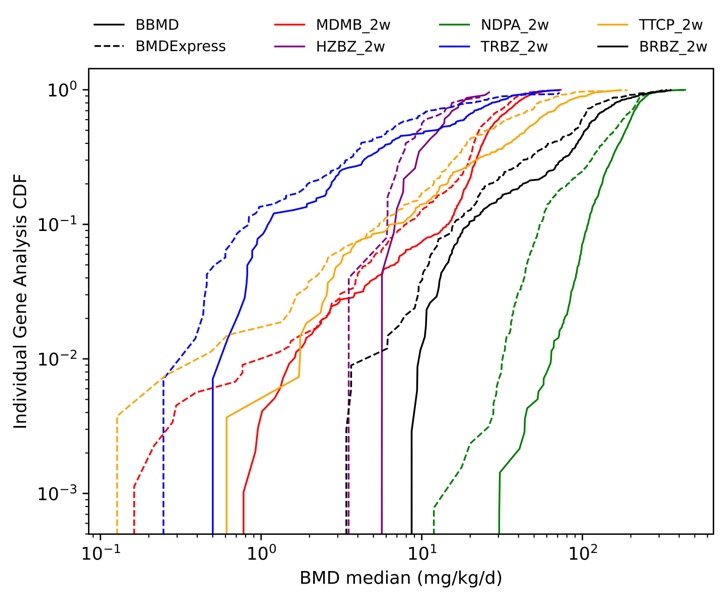


(c) (d)


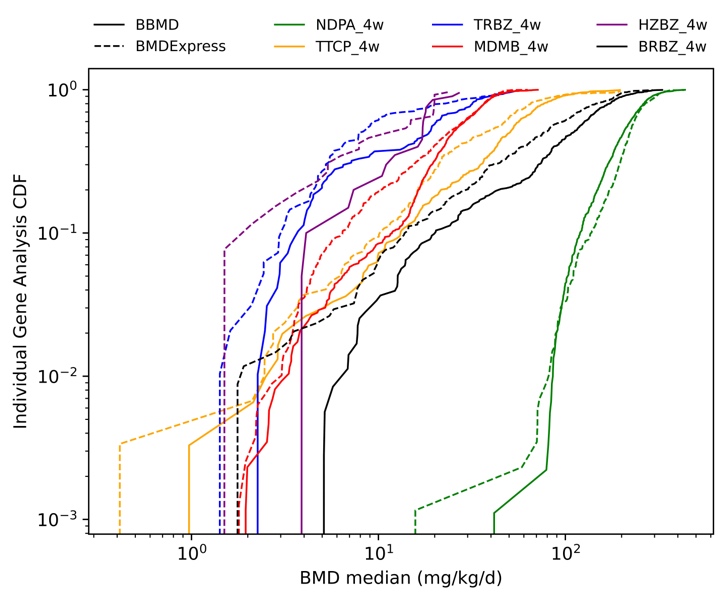

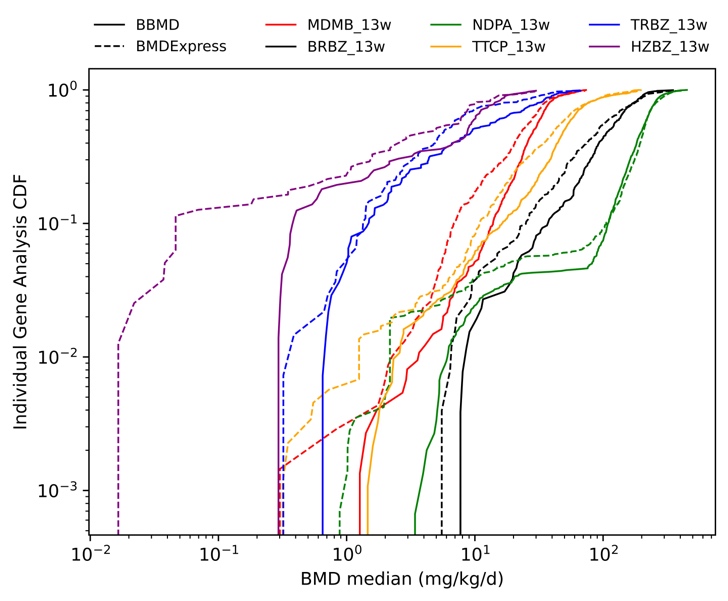


Figure S24 Log scaled cumulative distribution comparison of individual gene analysis for BRBZ, NDPA, TTCP, MDMB, TRBZ and HZBZ at (a) 5 days, (b) 2 weeks, (c) 4 weeks, and (d) 13 weeks (dashed lines are values from BMDExpress and solid lines represent values of BBMD)

- GO analysis comparison

(a) (b)


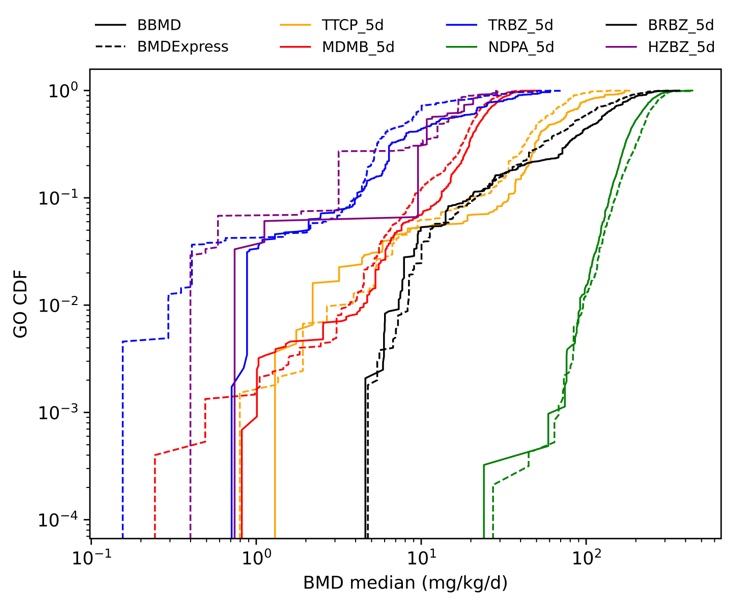

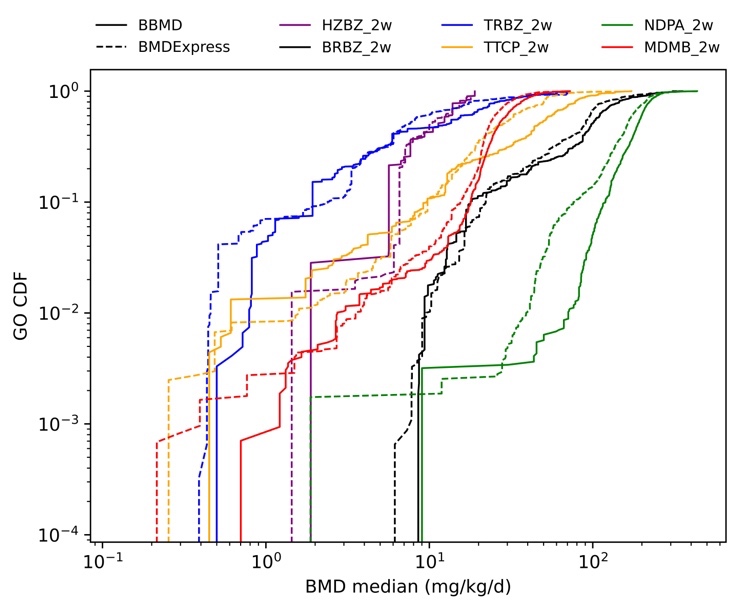


(c) (d)


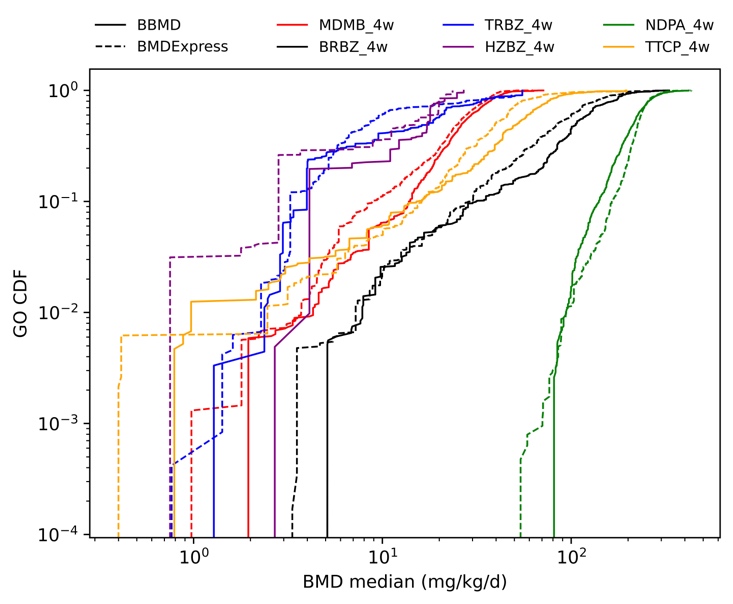

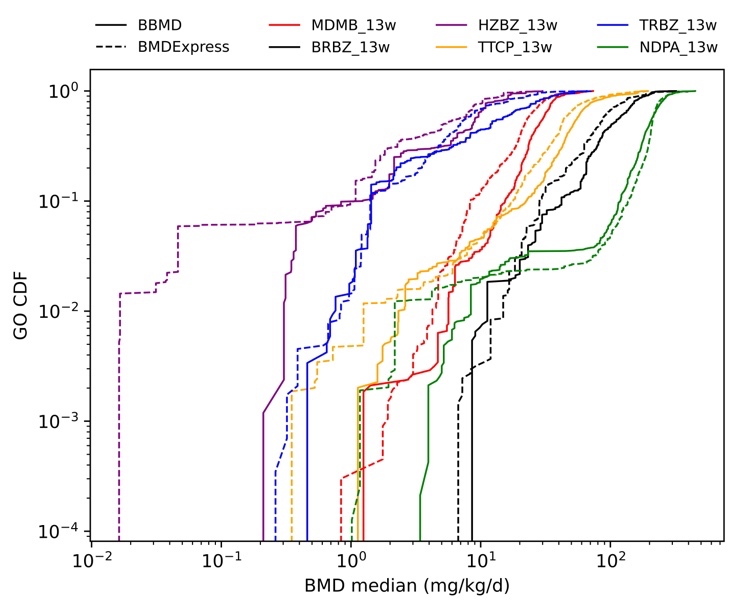


Figure S25 Log-scaled cumulative distribution comparison of GO analysis for BRBZ, NDPA, TTCP, MDMB, TRBZ and HZBZ at (a) 5 days, (b) 2 weeks, (c) 4 weeks, and (d) 13 weeks (dashed lines are values from BMDExpress and solid lines represent values of BBMD)

- REACTOME pathway analysis comparison

(a) (b)


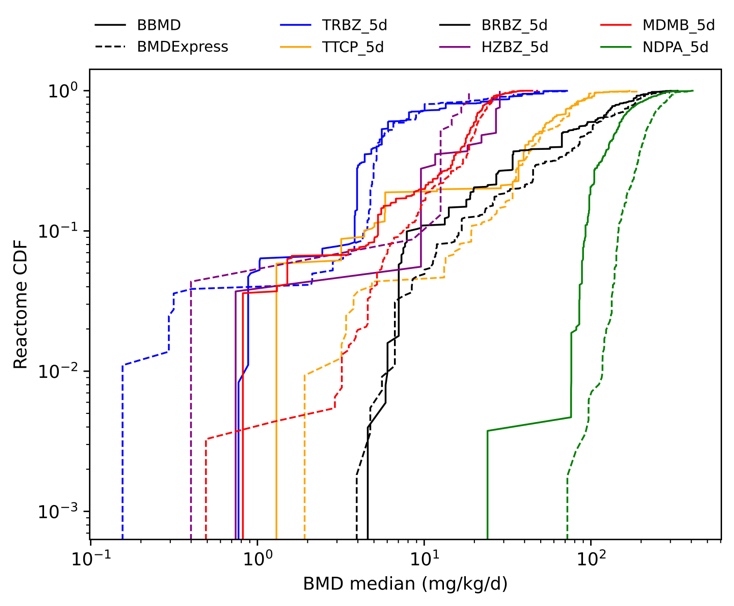

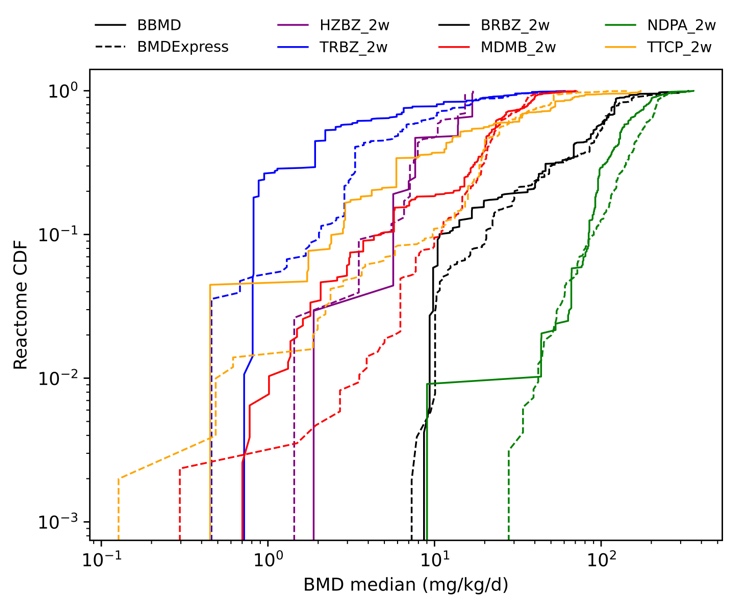


(c) (d)


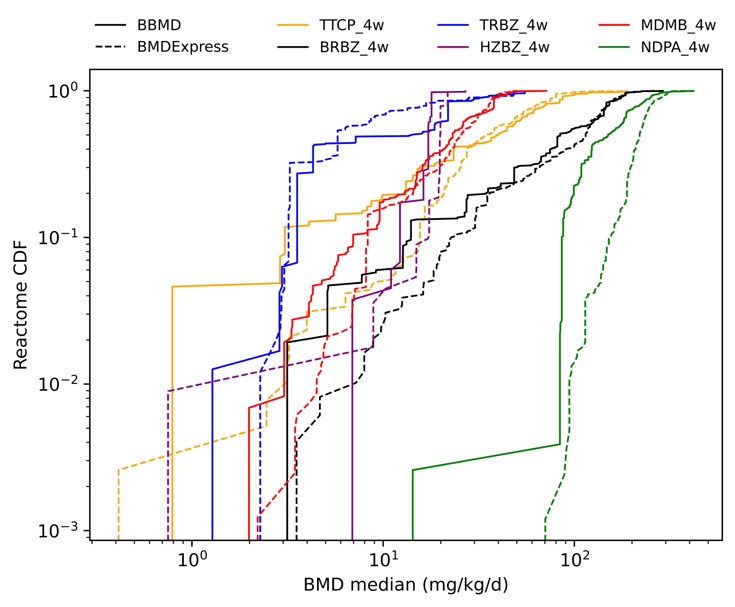

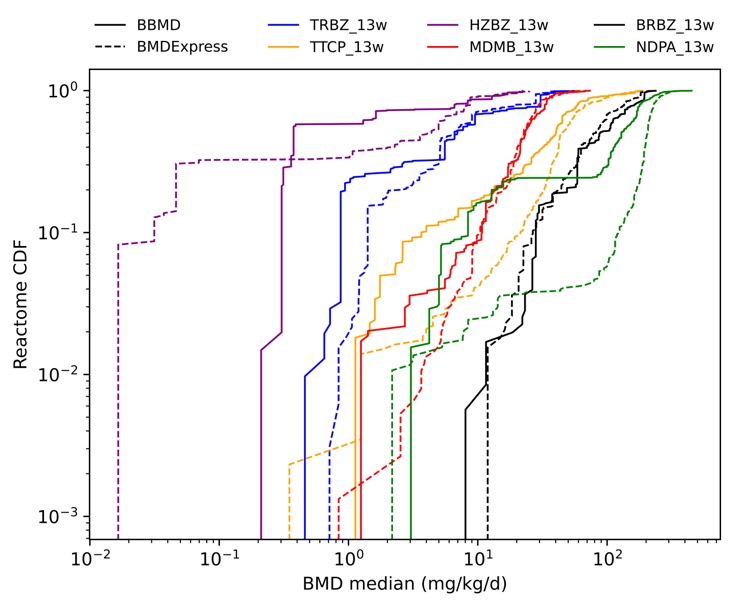


Figure S26 Log-scaled cumulative distribution comparison of REACTOME pathway analysis for BRBZ, NDPA, TTCP, MDMB, TRBZ and HZBZ at (a) 5 days, (b) 2 weeks, (c) 4 weeks, and (d) 13 weeks (dashed lines are values from BMDExpress and solid lines represent values of BBMD

**References**

Thomas RS, Wesselkamper SC, Wang NCY, Zhao QJ, Petersen DD, Lambert JC, et al. 2013. Temporal concordance between apical and transcriptional points of departure for chemical risk assessment. Toxicological Sciences 134:180-194.

Johnson KJ, Auerbach SS, Costa E. 2020. A rat liver transcriptomic point of departure predicts a prospective liver or non-liver apical point of departure. Toxicological Sciences 176:86-102.
